# Performance of Zr-Based Metal–Organic Framework Materials as In Vitro Systems for the Oral Delivery of Captopril and Ibuprofen

**DOI:** 10.3390/ijms241813887

**Published:** 2023-09-09

**Authors:** Carmen Cretu, Roxana Nicola, Sorin-Alin Marinescu, Elena-Mirela Picioruș, Mariana Suba, Corina Duda-Seiman, Adel Len, Levente Illés, Zsolt Endre Horváth, Ana-Maria Putz

**Affiliations:** 1 “Coriolan Drăgulescu” Institute of Chemistry, Bv. Mihai Viteazu, No. 24, 300223 Timisoara, Romania; carmencretu78@gmail.com (C.C.); cc.roxana@yahoo.com (R.N.); sorin.alin.marinescu@gmail.com (S.-A.M.); mirela_taerel82@yahoo.com (E.-M.P.); marianasuba@gmail.com (M.S.); 2 Biology-Chemistry Department, West University of Timisoara, Johann Heinrich Pestalozzi No. 16, 300115 Timisoara, Romania; corina.seiman78@e-uvt.ro; 3 Institute for Energy Security and Environmental Safety, Centre for Energy Research, Konkoly-Thege Miklós Út 29-33, 1121 Budapest, Hungary; adel.len@ek-cer.hu; 4 Faculty of Engineering and Information Technology, University of Pécs, Boszorkány Street 2, 7624 Pécs, Hungary; 5 Institute for Technical Physics and Material Science, Centre for Energy Research, Konkoly-Thege Út 29-33, 1121 Budapest, Hungary; illes.levente@ek-cer.hu (L.I.); horvath.zsolt.endre@ek-cer.hu (Z.E.H.)

**Keywords:** Zr-MOF, solvothermal, captopril, ibuprofen, in vitro oral delivery

## Abstract

Zr-based metal–organic framework materials (Zr-MOFs) with increased specific surface area and pore volume were obtained using chemical (two materials, **Zr-MOF1** and **Zr-MOF3**) and solvothermal (**Zr-MOF2**) synthesis methods and investigated via FT-IR spectroscopy, TGA, SANS, PXRD, and SEM methods. The difference between **Zr-MOF1** and **Zr-MOF3** lies in the addition of reactants during synthesis. Nitrogen porosimetry data indicated the presence of pores with average dimensions of ~4 nm; using SANS, the average size of the **Zr-MOF** nanocrystals was suggested to be approximately 30 nm. The patterns obtained through PXRD were characterized by similar features that point to well-crystallized phases specific for the UIO-66 type materials; SEM also revealed that the materials were composed of small and agglomerate crystals. Thermogravimetric analysis revealed that both materials had approximately two linker deficiencies per Zr_6_ formula unit. Captopril and ibuprofen loading and release experiments in different buffered solutions were performed using the obtained Zr-based metal–organic frameworks as drug carriers envisaged for controlled drug release. The carriers demonstrated enhanced drug-loading capacity and showed relatively good results in drug delivery. The cumulative percentage of drug release in phosphate-buffered solution at pH 7.4 was higher than that in buffered solution at pH 1.2. The release rate could be controlled by changing the pH of the releasing solution. Different captopril release behaviors were observed when the experiments were performed using a permeable dialysis membrane.

## 1. Introduction

Metal–organic frameworks (MOFs), also known as porous coordination polymers, are constructed by linking inorganic and organic units through strong bonds, which can be connected into one-, two-, or three-dimensional lattices [1]. MOFs are a class of porous materials characterized by simple synthesis, facile structural modification, and various potential applications [2,3]. Owing to their remarkable porosity, chemical and thermal stability, and almost unlimited possibilities for designing targeted structures through metal-linker chemistry and post-synthetic modification [4,5], these materials are promising, especially for biomedical applications. Among the large families of MOFs, Zr-based MOFs (Zr-MOFs), which exhibit rich structural types, usually possess excellent chemical, thermal, and water stability owing to their strong Zr-O bonds and stable secondary building units [6]. Zirconium is a biocompatible metal (the human body contains approximately about 300 mg of zirconium, and recommended daily ingestion is 4.15 mg/day) [7]; therefore, interest in zirconium-based MOFs and functionalized Zr-MOFs for biomedical applications and drug delivery has increased [5]. In our previous study [8], we presented an investigation of the rapid modulated synthesis of micro-/meso-sized Zr-MOF, porous material known as UIO-66, containing terephthalic acid (H_2_BDC) as an organic linker using excess metal salt precursor (ZrOCl_2_·8H_2_O) and different concentrations of acetic acid (13 Eq, 26 Eq, 52 Eq, and 104 Eq) as organic modulators. The term Eq = equivalent refers to the molar ratio of acetic acid to ZrOCl_2_·8H_2_O in a typical synthesis procedure. Many studies have demonstrated the effects of different modulators on MOF structures, resulting in an increase in crystallinity, as well as missing cluster or linker defects [9,10,11], with a positive impact on gas adsorption or drug delivery [12]. The materials obtained with 26 Eq of acetic acid (by activation in EtOH) were chosen as the starting recipe, because a moderate surface area of 703 m^2^/g with a total pore volume of 1.16 cm^3^/g was obtained [8]. In this study, new materials were prepared using methanol instead of ethanol as the solvent during the activation process. To be applicable as drug delivery systems, Zr-MOF materials must fulfil certain requirements: biocompatibility and an ordered pore network, narrow size distribution, and high surface area. Hybrid aerogel microparticles as drug delivery systems for cervical cancer chemotherapy can prevent extravasation [13] or remove protein-bound uremic toxins from blood, such as in the case of ibuprofen loaded onto metal–organic framework shells on Fe_3_O_4_ nanoparticles [14].

**Figure 1:**
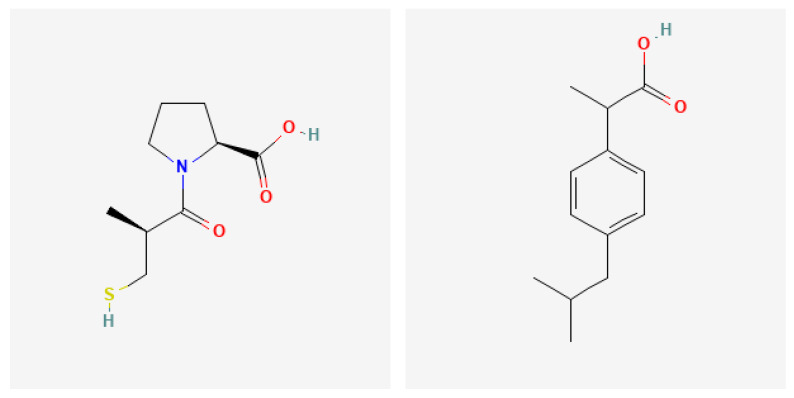
Chemical formulae of captopril (**left**) [15] and ibuprofen (**right**) [16].

Captopril (CP) [15] and ibuprofen (IBU) [16] were chosen as model drugs; their structures are shown in Figure 1. Captopril ((2S)-1-[(2S)-2-methyl-3-sulfanylpropanoyl] pyrrolidine-2-carboxylic acid) is a water-soluble drug with a molecule size of 9.0 × 5.7 × 3.3 Å, characterized by the presence of a sulfhydryl group and its active region, the carboxyl group. Captopril is an angiotensin-converting enzyme inhibitor drug [17]. Captopril is highly water-soluble and has an elimination half-life of 1.7 h after oral dose [18]. It is usually prescribed to patients who are chronically ill and require long-term therapeutic benefits. The development of a once-daily captopril oral formulation would be a significant advantage for patient compliance, accompanied by the minimization of drug side effects as a result of reducing the concentration fluctuation of the drug in the blood, especially in the case of long-term therapy [19,20]. Ibuprofen (2-(4-Isobutylphenyl)propanoic acid) is a nonsteroidal anti-inflammatory drug (NSAID) with analgesic and antipyretic effects [21]. IBU has a molecular size of ~10.3 × 5.3 Å and is commonly used as a model drug for delivery [22,23]. The typical therapeutic range of IBU in human blood is approximately 10–50 mg/L, and its toxicity level is greater than 250 mg/L [22,23]. Ibuprofen is a relatively weak acid with a pKa value of 4.4. It has low solubility in water (0.06 mg/mL) or at acidic pH, and is soluble in ethanol, but its solubility increases steeply with pH. Therefore, the drug is largely insoluble at low pH, but becomes soluble at alkaline pH [24]. To avoid these drawbacks, solid carriers can be used to improve the therapeutic efficacy and reduce the side effects of the drug [25]. These solid carriers can enhance contemporary antibacterial treatment for chronic wounds [26]. The tunable structure and high porosity of Zn-based MOFs facilitate the loading of biological molecules [27]. Polyrhodanine-stabilized Fe_3_O_4_/graphene oxides can act as drug carriers because of their low toxicity and may open a new window for antibacterial agents [28]. Recently, polythiophene-based compounds prepared using nanocomposites to facilitate drug delivery have also been developed [29]. Multifunctional gold nanorods have been developed for therapeutic applications and pharmaceutical delivery considering cellular metabolic responses, oxidative stress and cellular longevity [30]. Bioactive graphene quantum-dot-based polymer composites have a high specific surface area and high drug loading capacity and can be used as an excellent option for the release of anticancer drugs [31].

In the present study, Zr-based metal–organic framework materials (Zr-MOFs) with increased specific surface areas and total pore volumes were obtained using chemical and solvothermal synthesis methods. Captopril and ibuprofen loading and release experiments were performed in different buffered solutions using the obtained Zr-based MOF as drug carriers.

## 2. Results

### 2.1. FT-IR

**Figure 2:**
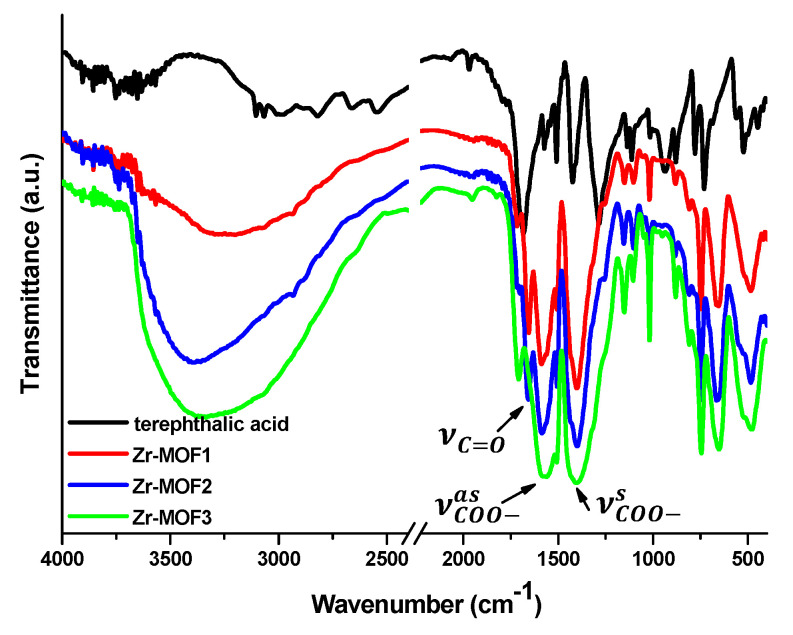
FT-IR spectra of **Zr-MOF1-3** samples compared to terephthalic acid.

The FT-IR spectra recorded for the three Zr-MOF materials (Figure 2) are in agreement with data reported in the literature for UIO-66 MOF [32]; new bands found at 1580 cm^−1^ and 1400 cm^−1^, respectively, in the **Zr-MOF1–3** spectra can be attributed to the asymmetric and symmetric stretching vibrations of the coordinated carboxylate groups. These bands appear shifted towards lower frequencies compared to of those the free ligand. Moreover, new bands assigned to the carbonyl group (C=O) of the DMF residues at 1660 cm^−1^ and ZrO in the range 660–450 cm^−1^ were present in the MOF’ spectra [33].

### 2.2. Thermal Stability

**Figure 3:**
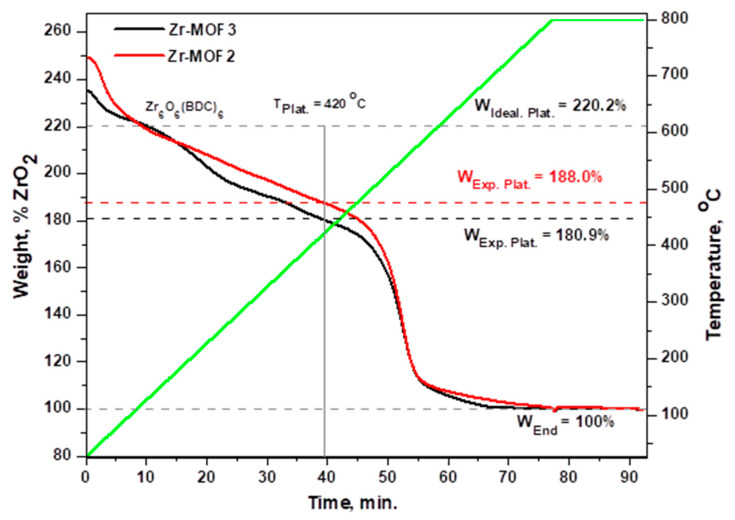
**Zr-MOF2** and **Zr-MOF3** thermogravimetric (TGA) curves normalized to 100% ZrO_2_. Vertical line, **T_Plat._** is the temperature at which the plateaus (**W_Exp.Plat_**_._) were obtained for each sample.

Thermogravimetric analysis of **Zr-MOF2** and **Zr-MOF3** (Figure 3 and Figure S4) showed a steady decrease in mass up to 420 °C, consisting of the loss of adsorbed water molecules (25–100 °C), loss of residual DMF trapped inside the pores and structural water (150–300 °C) and loss of acetic acid (300–400 °C). Above 420 °C, decomposition begins with BDC linker loss. To ensure the complete combustion of the organic fragments, dynamic air flow was introduced at 800 °C to obtain a ZrO_2_ residue. Although the materials were activated and dried under vacuum, DMF was not entirely removed from the pores. Moreover, during cooling to room temperature after drying, the samples adsorbed atmospheric water. To calculate the number of linker deficiencies in the prepared materials, we normalized the end weight of the TGA to 100% ZrO_2_. The calculations were performed with respect to the ideal UiO-66 sample following the method described by Shearer et al. [11]. The plateau for each sample was chosen as the temperature at which everything except the BDC linker was lost (Supplementary Material Figure S4). We observed that the experimental plateaus for **Zr-MOF2** and **Zr-MOF3** were lower than the plateau of the UIO-66 samples (defect–free), suggesting a linker deficiency in our materials. The ideal UiO-66 sample had a composition of Zr_6_O_6_(BDC)_6_, and the defective UiO-66 sample had a Zr_6_O_6+x_(BDC)_6−x_ composition (where x represents the number of missing linkers per Zr_6_ formula unit). By applying the equations described by Shearer [11], we obtained a composition of Zr_6_O_7.61_(BDC)_4.39_ for **Zr-MOF2** and Zr_6_O_7.97_(BDC)_4.03_ for **Zr-MOF3**. Both materials have approximately two linker deficiencies per Zr6 unit.

### 2.3. Textural Characterization of Zr-MOFs by Nitrogen Sorption Method

The textural parameters of the obtained materials are listed in Table 1. Following the same recipe described in our previous study [8], in the present study a new **Zr-MOF1** material was obtained with a better surface area, 1123 m^2^/g (with 80.5% microporosity fraction), than the previously reported (703 m^2^/g) and an almost similar total pore volume of 1.06 cm^3^/g (1.16 cm^3^/g). For the new materials synthetized in this study, methanol was used instead of ethanol as the solvent in the activation process. In the case of **Zr-MOF2** obtained by the solvothermal method and **Zr-MOF3** obtained by reordering the steps of adding the reactants, unexpected lower surface areas of 912 m^2^/g (with a 74.34% fraction of microporosity) and 731 m^2^/g (with a 65.94% fraction of microporosity), respectively, with a better pore size distribution than **Zr-MOF1**, taking into consideration the linker deficiencies found in these Zr-MOFs. However, this can be explained by the fact that the pores were filled with re-adsorbed water vapor and residual solvent. The highest fraction of micropores has been calculated for the **Zr-MOF1** sample, 904 m^2^/g for the micropore surface area and 0.38 cm^3^/g for the total micropore volume.

**Figure 4:**
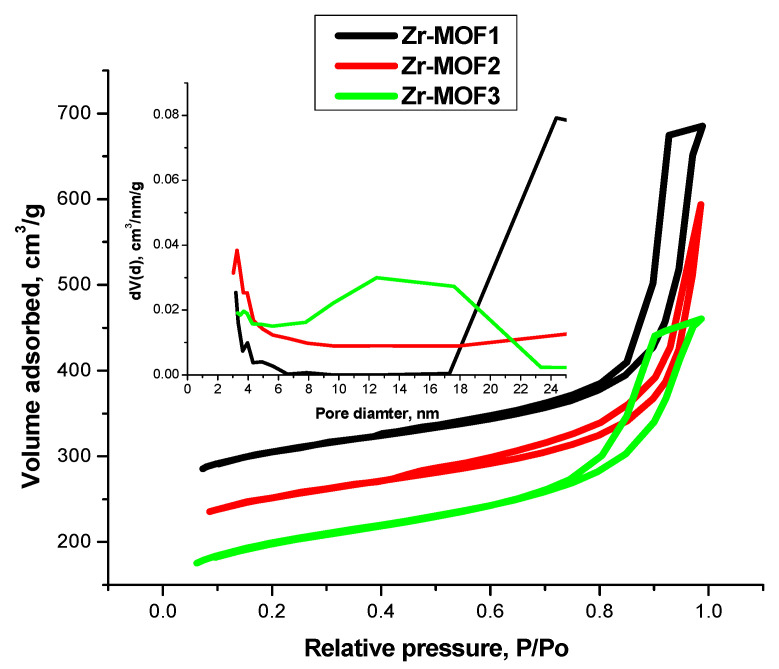
Adsorption–desorption isotherms and pore size distribution of the obtained **Zr-MOFs**: **Zr-MOF1** (used for captopril), **Zr-MOF2** (used for ibuprofen), and **Zr-MOF3** (used for the dialysis release procedure).

Figure 4 presents the adsorption–desorption isotherms and pore size distributions for all the synthetized samples. All samples presented Type IV isotherms specific for mesoporous materials, with H2b type hysteresis loops for **Zr-MOF1** and **Zr-MOF3**, characteristics of pores with larger neck widths associated with pore blocking, and H1 type hysteresis loop for **Zr-MOF2**, characteristics for ink-bottle pores [34]. The pore size distribution (Figure 4 inset) is a wider unimodal distribution, with a majority of the pores being around 3–4 nm.

### 2.4. SANS (Small-Angle Neutron Scattering)

**Figure 5:**
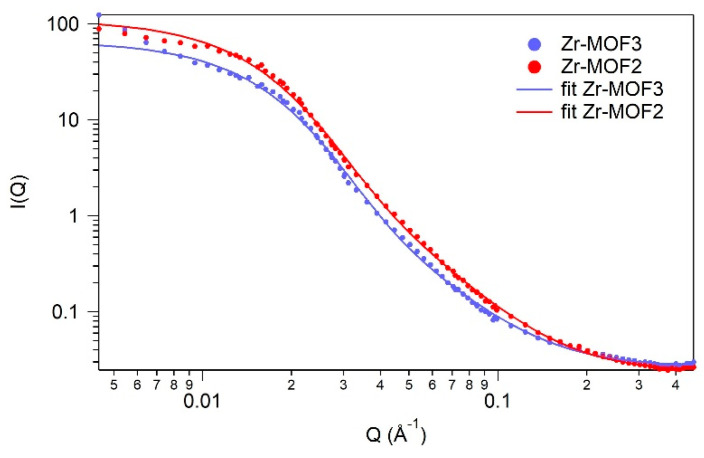
SANS profiles of **Zr-MOF2** and **Zr-MOF3** samples.

The recorded two-dimensional scattering intensities were radially averaged, and after the regular calibration procedure (taking into account the transmission of the sample, scattering from the quartz holder, background noise, and detector pixel sensitivity), the intensity versus scattering vector (Q=4π sinθ/λ, where *λ* is the wavelength and *θ* is half of the scattering angle) curves were plotted (Figure 5).

The available Q range (0.005–0.4 ${Å}^{-1}$) is applicable for probing scattering objects (pores, grains, crystallites) between ~15÷1000 Å. Using a standard procedure, the shape and size of these objects were modelled mathematically, and the least squares fitting method was used to find the most adequate model that describes the nanomorphology.

In the studied Q range, the **Zr-MOF2** and **Zr-MOF3** samples showed similarly shaped scattering curves, which led us to conclude that in the nanometric size range, the morphology of the samples does not depend on the reaction conditions used for their preparation.

The best fit was obtained by the first-level Beaucage model [35], which unifies the shape-independent Guinier and power-law approximations. The unified model (Equation (1)) is applicable to describe the entire measured Q range; therefore, it provides a characteristic average size of the scattering objects and a power-law exponent describing the fractal behavior of the system.

(1) $$I\left(Q\right)=A\;exp\left(-\frac{Q^{2}Rg^{2}}{3}\right)+B\frac{{\left[\mathrm{erf}(QRg/\sqrt{6})\right]}^{3p}}{Q^{p}}$$

where *Rg* is the gyration radius, *p* is the power-law exponent, and *A* and *B* are coefficients related to the volume and number density of the scattering objects and their contrast. Data fitting was performed using the SANS algorithm [36] created for the nonlinear least-squares fitting method using Igor Pro 6.1 software.

The calculated gyration radii for **Zr-MOF2** and **Zr-MOF3** were 121 Å and 127 Å, respectively, which represent average diameters of 31 nm and 33 nm of the scattering objects. The nitrogen porosimetry data indicated the presence of pores with an average dimension of ~4 nm; therefore, a size of approximately 30 nm is suggested to be the average size of Zr-MOF nanocrystals [37]. The value of the p exponential parameter was calculated as 2.6 and 2.8 for the two specimens, which characterizes a mass fractal behavior [38]. Mass fractals in SANS can be understood as 3D structures with subunits, such as clusters of pre-MOF structures [37].

### 2.5. X-ray Diffraction

**Figure 6:**
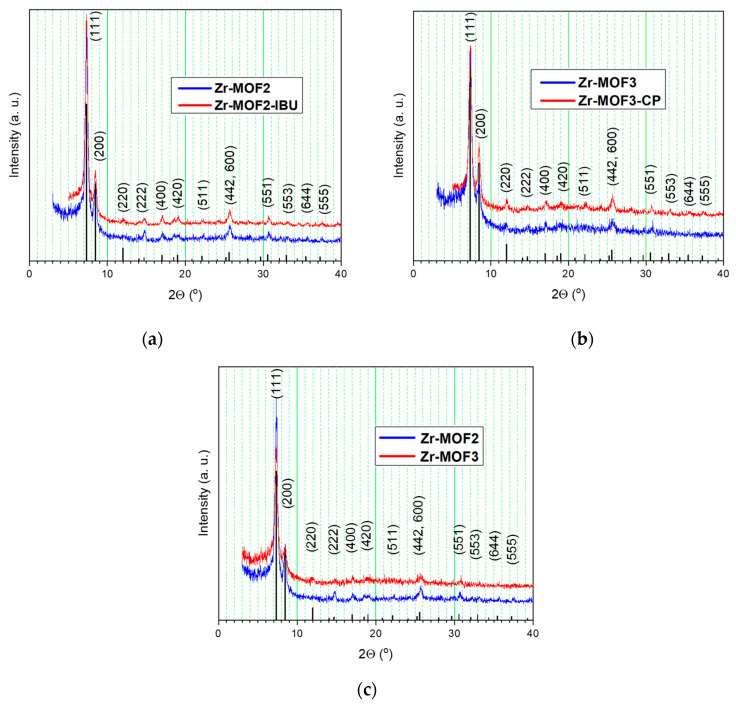
XRD patterns for **Zr-MOF2** and after drug loading **Zr-MOF2-IBU** (**a**); **Zr-MOF3** and after drug loading **Zr-MOF3-CP** (**b**); XRD patterns for **Zr-MOF2** and **Zr-MOF3** (**c**).

The X-ray diffraction (XRD) patterns of the synthesized samples are shown in Figure 6. The line positions were calculated using the theoretical structure from Øien S et al. [39], using VESTA software 3.5.7 [40]. Miller indices are presented. These patterns are characterized by similar features that point to well-crystallized phases specific to the UIO-66 type materials [41]. **Zr-MOF2** displayed higher XRD peak intensities than **Zr-MOF3** (Figure 6c), because of the solvothermal synthesis conditions used for **Zr-MOF2** preparation. The materials did not lose their crystallinity after drug loading, in both cases with IBU (Figure 6a) and CP (Figure 6b). The drug-loading process did not alter the crystalline structure of the porous materials. The absence of Bragg peaks corresponding to free CP and IBU demonstrated that free drugs in significant quantities did not recrystallize outside the pores [41].

### 2.6. Scanning Electron Microscopy

**Figure 7:**
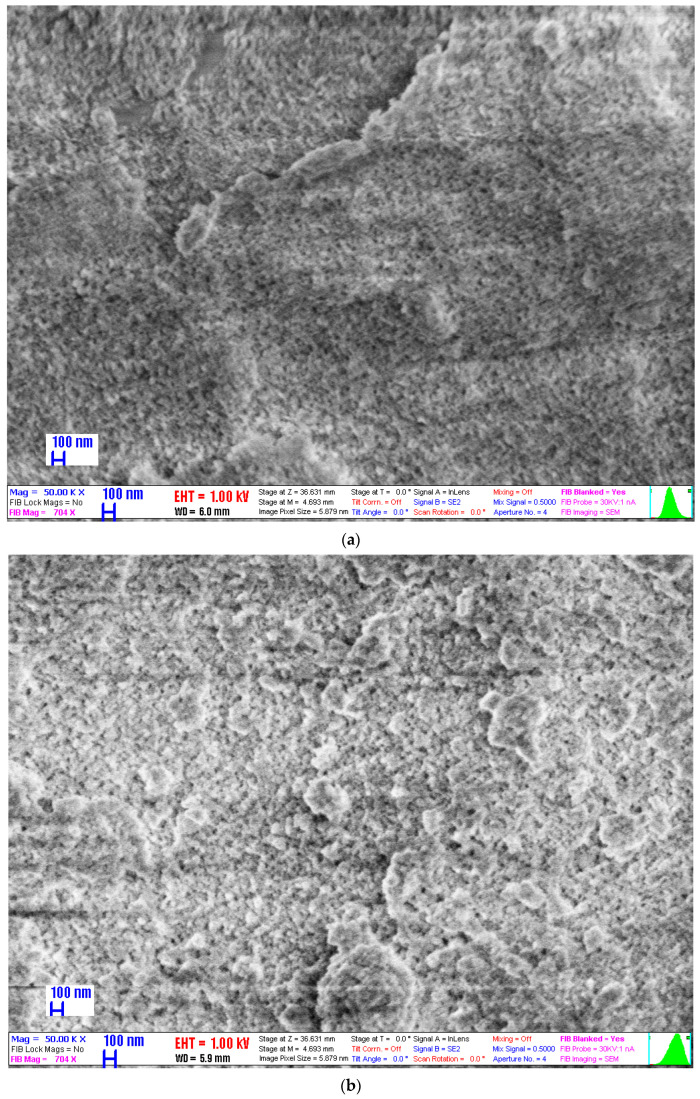

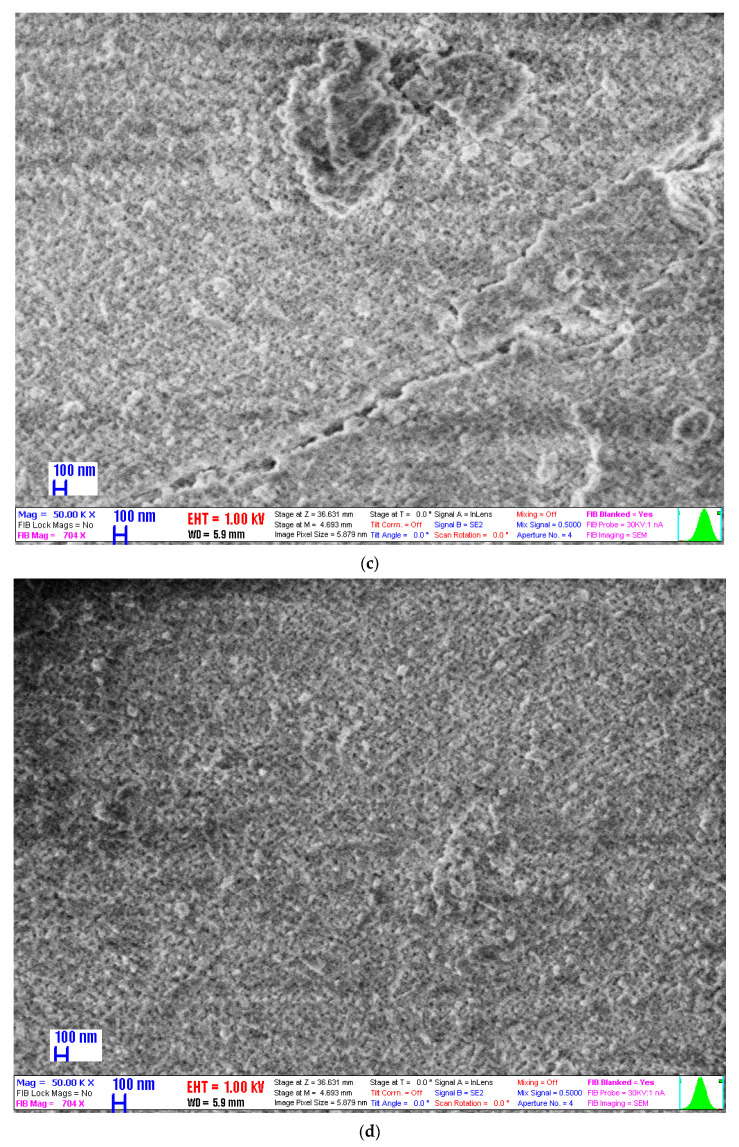
SEM images of the samples: (**a**) **Zr-MOF2**, (**b**) **Zr-MOF2-IBU**, (**c**) **Zr-MOF3** and (**d**) **Zr-MOF3-CP**.

The SEM images of the samples **Zr-MOF2**, **Zr-MOF2-IBU**, **Zr-MOF3** and **Zr-MOF3-CP** are presented in Figure 7. No substantial differences were observed in the morphology of the samples after drug loading. The materials were composed of small and agglomerated crystals (the crystallinity of the obtained materials was confirmed by XRD, see Figure 6), and the elemental composition of the samples was studied by energy dispersive X-ray spectrometry (EDX); the results are presented in Supplementary Materials Figure S5.

### 2.7. Drug Loading

The loading efficiency (%) and loading capacity (mg/g) (the results are presented in Table 2) were calculated for each material entrapment in each experiment using the following formulas:

(2) $$\mathrm{Loading}\;\mathrm{Efficiency}\;\left(\%\right)=\left(\mathrm{Total}\;\mathrm{drug}\;\mathrm{added}-\mathrm{free}\;\mathrm{non}\;\mathrm{entraped}\;\mathrm{drug}\right)\times \frac{100}{\mathrm{Total}\;\mathrm{drug}\;\mathrm{added}}$$

(3) $$\mathrm{Loading}\;\mathrm{Capacity}\;\left(\frac{\mathrm{mg}}{g}\right)=\frac{\left(\mathrm{Total}\;\mathrm{drug}\;\mathrm{added}-\mathrm{free}\;\mathrm{non}\;\mathrm{entraped}\;\mathrm{drug}\right)}{\mathrm{Amount}\;\mathrm{of}\;\mathrm{carrier}\;}$$

### 2.8. Drug Release

#### 2.8.1. Captopril Release from **Zr-MOF1-CP** Sample Material Synthetized Using the Chemical Method

**Figure 8:**
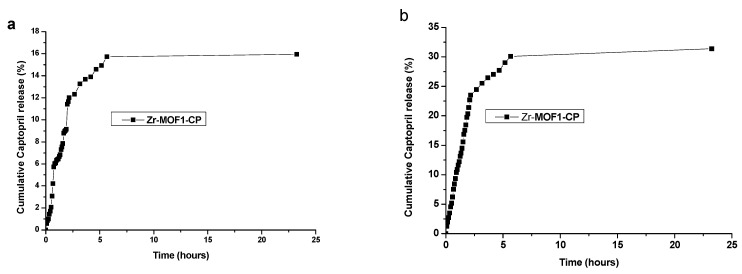
The percentage of cumulative captopril release in time for the **Zr-MOF1-CP** sample in (**a**) hydrochloric acid solution (pH = 1.2) and (**b**) phosphate-buffered solution (pH = 7.4).

For the **Zr-MOF1-CP** sample, in acidic buffer solution (simulated stomach media), 15.7% of captopril was released after 5.6 h; this release was only slightly increased to 15.9% after 23.25 h (Figure 8a). In phosphate-buffered solution (simulated intestine media), 30.08% of the captopril was released after 5.6 h; this release was only slightly increased to 31.38% after 23.25 h (Figure 8b). Table 3 presents the results.

#### 2.8.2. Ibuprofen Release from **Zr-MOF2-IBU**

**Figure 9:**
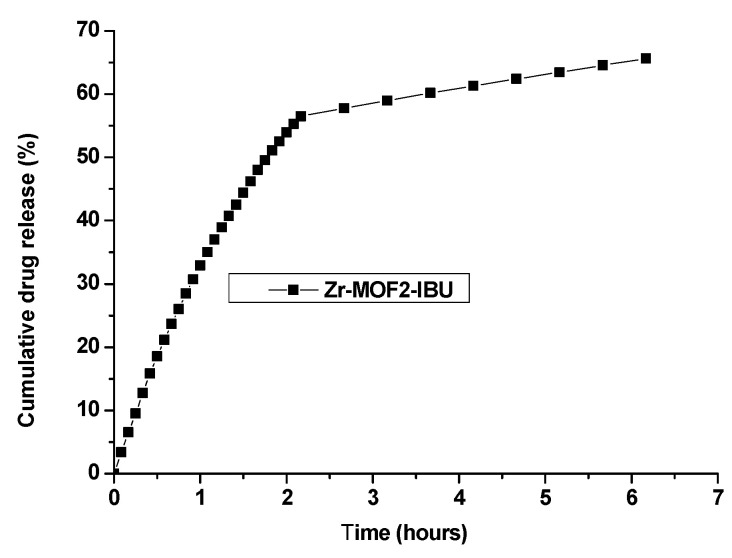
The percentage of ibuprofen released over time for the **Zr-MOF2-IBU** sample in phosphate-buffered solution (pH = 7.4).

For the **Zr-MOF2-IBU** sample, in a phosphate-buffered solution (simulated intestine media), 65.62% of the ibuprofen was released after 6.17 h (Figure 9 and Table 4).

#### 2.8.3. Captopril Release Using the Dialysis Membrane from **Zr-MOF3-CP**

**Figure 10:**
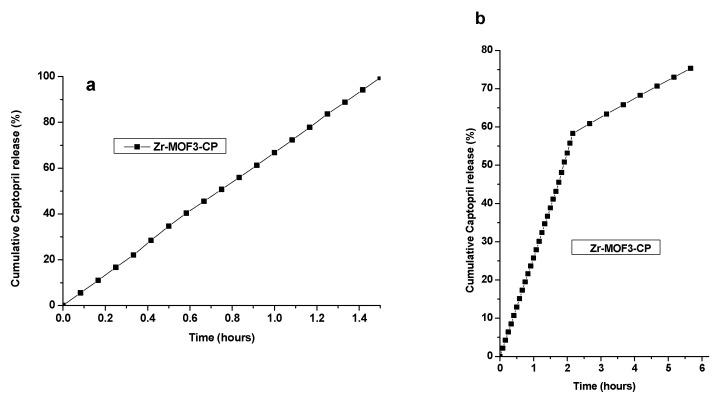
Cumulative captopril release over time for the **Zr-MOF3-CP** sample in (**a**) hydrochloric acid solution (pH = 1.2) and (**b**) phosphate-buffered solution (pH = 7.4) using a dialysis membrane.

For the **Zr-MOF3-CP** sample, in an acidic buffer solution (simulated stomachal media), 99.51% of the captopril was released after 1.5 h (Figure 10a). In a phosphate-buffered solution (simulated intestinal media), 75.29% of captopril was released after 5.6 h (Figure 10b). Table 5 presents the results.

### 2.9. Kinetics Models Applied for the First Hours of Release

The in vitro release data were applied to various kinetic models to predict drug release mechanisms and kinetics. The calculated parameters are presented in the Supplementary Material: Tables S1 and S2 (for captopril in HCl and phosphate buffers, respectively), Table S3 (for ibuprofen), and Tables S4 and S5 (for captopril using a dialysis membrane, in HCl and phosphate-buffered solution, respectively). Table S1: Calculated parameters for captopril release kinetic in acidic buffer. Table S2: Calculated parameters for captopril release kinetics in phosphate buffer. Table S3: Parameters calculated for the release kinetics of ibuprofen in phosphate buffer. Table S4: Parameters calculated for the release kinetics of captopril in acidic buffer using a dialysis membrane. Table S5: Parameters calculated for the release kinetics of captopril in phosphate-buffered solution using dialysis membranes.

The different kinetic models applied for drug release in different buffers are presented in Figure S1a–e (pH 1.2 buffer solution), in Figure S1f–j (pH 7.4 buffer solution) (for captopril), in Figure S2a–e (pH 7.4 buffer solution) (for ibuprofen), and Figure S3a–e (pH 1.2 buffer solution), in Figure S3f–j (pH 7.4 buffer solution) (for dialysis procedure with captopril).

For the Zero Order Kinetic Model, the data obtained from the in vitro drug release studies were plotted as the cumulative amount of drug released versus time. In the First Order Kinetic Model, the data obtained were plotted as the log cumulative percentage of the drug remaining versus time. This model is valid for both water-soluble drugs and burst-like release.

In the Higuchi model, data are represented as the cumulative percentage of drug release versus the square root of time. It defines the release of drugs based on their diffusion from an insoluble homogeneous matrix. This model is valid for all release types [42]. Korsmeyer–Peppas is a simple model known as “Power law” describing drug release from a polymer system. The Korsmeyer–Peppas model describes some release mechanisms simultaneously, such as the diffusion of water into the matrix, swelling of the matrix, and dissolution of the matrix. To study the release kinetics, data obtained from the in vitro drug release studies were plotted as log cumulative percentage drug release versus log time. This model is valid only for small values of the release time. For the Hixson–Crowell model, Hixson and Crowell (1931) discovered that a group of particles’ regular area is proportional to the cube root of its volume. To study the release kinetics, data obtained from in vitro drug release studies were plotted as cube root of drug percentage remaining, Wo-Wt (cube root of % drug remaining), in a matrix versus time [42].

The different kinetic models were fitted to the first hour or to the first 1.5 h or 5.67 h release data. The calculated coefficients of determination (*R*^2^) are listed in Tables S6–S8.

## 3. Discussion

The preparation method and working procedure of Zr-MOFs can generate materials with different surface areas and pore size distributions. Thus, following the chemical method, we obtained two materials (**Zr-MOF1** and **Zr-MOF3**) with surface areas of 1123 and 731 m^2^/g, respectively, and total pore volume of 1.06 and 0.71 cm^3^/g, depending on the work procedure. In the case of **Zr-MOF2** prepared by the solvothermal method, a medium surface area of 912 m^2^/g was obtained with an increased pore size distribution compared with the other two materials. The materials present well-crystallized phases specific for the UIO-66 type materials and are composed of agglomerate nanocrystals approximately 30 nm in size with pores of ~4 nm.

### 3.1. Drug Loading

Surface area is the most determining factor for the amount of adsorbed drug because the drug loading process is mainly based on the adsorptive properties of the material. Drug loading can be tuned by increasing or decreasing the surface area (once the pore size has allowed the drug to enter the matrix, the higher the surface area, the higher the quantity of adsorbed drug). In the present work, the high values of the specific surface area obtained for all three materials promoted them as materials with good drug adsorption properties. Concerning the pore size, the adsorption of drug molecules can also be tuned by size selectivity. Drug–pore interaction is a surface phenomenon; however, weak drug–drug interactions can also be present under loading conditions that can promote pore filling. Therefore, several consecutive loadings can lead to an increased amount of loaded drugs due to increased drug intermolecular interactions. In the present study, several consecutive loadings were not necessary because very good drug loading was achieved in the first loading step. The difference in pore size between our obtained Zr-MOF materials was small (the average pore size obtained between 3.78 and 4.04 nm), reducing the effect of the difference in pore size on drug loading, as previously reported in the literature [43].

In another study, three different drugs were added to a one-pot solvothermal synthesis (therefore, during the Zr-MOF synthesis procedure, not afterwards by adsorption, as in our case) and were distributed throughout the MOF at defect sites by coordination to the metal clusters, allowing a fourth drug to be post-synthetically loaded into the MOFs. The drugs become attached to the Zr clusters of the resulting MOFs, which has been found to be related to both the pKa of the metal-binding unit (the lower the pKa, the higher the incorporation) and its chemical functionality (phosphonates have a higher affinity for Zr than carboxylates and are incorporated more). As the drug-modulators are attached as defects rather than pore-loaded, the resultant carriers become highly porous, and they used their porosity to post-synthetically load another drug ultimately resulting in four drugs incorporated in significant quantities into a single nanovector. This means that the anticancer therapeutic activity of the double drug combinations towards MCF-7 breast cancer cells is highly increased for carriers containing the drugs compared with the free drugs, whereas the MOFs are biocompatible with to HEK293 kidney cells even at high doses, enhancing selectivity [44]. The concept of defect loading of drugs that act as modulators during synthesis can be applied to any therapeutic molecule containing carboxylate groups, such as doxorubicin [45]. Cisplatin prodrugs containing axial dichloroacetic acid ligands have been reported to be more effective than cisplatin and can overcome cisplatin resistance; similarly, dichloroacetic acid is known to enhance the anticancer effect of 5-fluorouracil and reduce resistance [45].

### 3.2. Drug Release

The strength of the specific interactions of the drug molecules and different carriers [46,47] realized via hydrogen bonds [48] could explain the low cumulative captopril release observed in the present work (as well as in our previous study when silica was used as a carrier [49]).

The hydrophilic drug–hydrophilic material interaction is stronger than the hydrophilic drug–hydrophobic material interaction; therefore, a more hydrophobic carrier extensively limits the cumulative release of captopril [50]. On the other hand, ibuprofen is less hydrophilic than captopril; therefore, a reduced release may be expected compared with captopril.

At an acidic pH, the captopril drug is protonated, and in the phosphate-buffered solution it is deprotonated [21,22]. Ibuprofen exists in neutral form at pH 4, the neutral/anionic form at pKa, and anionic form at pH 7 [51].

The remarkably extensive pH range that a Zr-MOF can sustain has been demonstrated in the literature; therefore, the MOF remains intact in aqueous solutions with pH values ranging from 1 to 11 [52]. Therefore, the Zr-MOF carrier exhibits a different behavior than silica. The rate of ibuprofen release can also be explained by interactions such as H-bonding (in small pores) and electrostatic repulsion [23] as in the case of functionalized Zr-MOF materials. The ibuprofen release rate in silica-containing materials has been described to decrease with decreasing pore size [53] and pH [54]; therefore, as previously mentioned, a different behavior arises as carriers between silica and Zr-MOFs.

In the buffer solution with mediation of cations, Coulomb interactions and ion pair formation strong interactions between the carrier matrices and drug molecules were expected to arise in the buffer solution at pH 7.4, in which could lead to a limited cumulative drug release [49], which is also valid for the Zr-MOF carrier.

MOFs are generally presented as crystalline powders. The crystal structure of UiO-66 has a six-center octahedral zirconium oxide unit, which is composed of octahedral Zr_6_O_4_(OH)_4_ units and terephthalate (1,4-benzene dicarboxylate (BDC)) linkers; each octahedral unit is connected to 12 adjacent units via BDC linkers, forming an expanded face-centered-cubic (FCC) structural unit. The high level of topological connectivity and strong coordination bonds between zirconium and oxygen collectively ensure the outstanding water stability of UiO-66, even under acidic or weakly alkaline conditions [55].

MOFs are constructed via the coordination assembly of metal ions/clusters and organic linkers, and provide a potential platform for drug delivery and controlled release to enhance therapeutic efficacy. However, more attention has been paid to the development of MOF-based drug-delivery systems in clinical area [56]. The drug-delivery experiment illustrated that the Camptothecin, an anticancer drug, release process contains three stages [56]. The organic ligands and particle sizes of Zr-based MOFs, such as Camptothecin molecules from the surface and from channel of the MOF-based nanocarriers, are important factors that affect the properties of drug loading, biocompatibility and toxicity [56,57]. The stability of the MOF in a PBS buffer (pH 7.4) was investigated by UV-Vis spectroscopic determination of fumarate release, showing that the smaller sample was initially more stable, but reached similar levels of linker release after 24 h. While Zr-MOF (with fumarate) degraded exponentially, reaching a plateau of 58% linker release after 8 h, the smaller analog degraded with a two-step profile, with a slower rate during the first 3 h (releasing 20% vs. 45% for the larger analog). Zr-MOF with fumarate seems to be more stable towards phosphate-induced degradation than Zr-MOF with terephthalate, which releases ~80% of its linker after a few hours. This might be a possible consequence of the lower pKa of fumaric acid compared with terephthalic acid, resulting in a more stable metal–carboxylate bond, thus enhancing the competition between free phosphates and carboxylates for coordination to the Zr clusters [58].

Lanthanide (thulium)-based MOFs have been used for the first time for selective removal of CO_2_ impurities from C_2_H_2_ (with an uptake of 5.83 mmol g^−1^ at 298 K and 1 bar) [59]. Bimetallic nanoparticles (Fe^2+^ + Zn^2+^)-doped ZIF-8/doxorubicin loaded (58.01% loading efficiency), resulting in nanocomposites (pH sensitive) for ferroptosis, have been used in the treatment of breast cancer. Ferroptosis, which induces the generation of reactive oxygen species in cancer cells, represents a promising strategy for cancer treatment [60]. Hf(IV)-based MOFs have also been tested in clinical cancer therapy using radiotherapy, chemotherapy, immunotherapy, phototherapeutic techniques, or a combination of two or more of these techniques [61]. MOFs have been used as carriers for the controlled delivery of Pt anticancer drugs in the form of metal complexes that can effectively solve tumor resistance [62]. Four mechanisms, including π-π interactions, Lewis acid/base complexing, hydrogen bonding, and anion-π interactions, were simultaneously involved in the adsorption by the MOFs: ibuprofen and naproxen to UiO-66 and UiO-66-NH_2_. Greater adsorption of ibuprofen onto MOFs was observed compared to naproxen, which is sustained by its higher binding energies with adsorbents. The binding energies followed the order π-π > hydrogen bonding > Lewis acid/base complexing > anion-π. The decreased adsorption of drugs with increasing pH was induced by the facilitated aggregation of MOFs at pH < pHpzc and the electrostatic repulsion between drugs and MOFs at pH > pHpzc. UiO-66 demonstrated much higher equilibrium adsorption amounts to ibuprofen (127.1 mg/g) and naproxen (88.51 mg/g) than UiO-66-NH_2_ (50.69 mg/g for ibuprofen and 40.10 mg/g for naproxen) [63]. Successful quantification of ibuprofen loading in UiO-66-NH_2_ was achieved based on the aerosol particle mass of MOF ≈ 55 mg of ibuprofen/g of UiO-66-NH_2_. The structural stability of UiO-66-NH_2_ versus ibuprofen release was successfully quantified over a 7-day period in an acidic phosphate-buffered solution, which provides a proof-of-concept scheme for controlled release studies of different types of active pharmaceutical ingredients from a variety of MOF-based nanocarrier systems [64]. The adsorptive removal of ibuprofen from a binary MOF (UiO-66 with 5% HKUST-1) and amine-functionalized UiO-66 in the aquatic environment: synergistic/antagonistic evaluation BET results showed that the binary MOF and UiO-66-NH_2_ had a smaller surface area and were mesoporous compared with UiO-66, while UiO-66 was microporous. UiO-66 (213 mg/g) had the highest adsorption among the adsorbents. UiO-66-NH_2_ showed the lowest adsorption (96 mg/g) due to a large decrease in the surface area. Despite its high surface area (1277.6 m^2^/g), the binary MOF had lower adsorption than UiO-66 (147 mg/g) because of the antagonistic effects between the adsorbent and ibuprofen. Additionally, increasing the pH above 4 reduced the adsorption of ibuprofen [65]. MOF/basswood-based nanocomposite molecularly imprinted membranes were smoothly compounded on the surface of three-dimensional (3D) basswood materials. Metal–organic framework (MOF) UiO-66-based particles were uniformly encapsulated in 3D basswood membranes by pretreating basswood with sodium hydroxide (NaOH). Ibuprofen was used as the template molecule throughout the dual-imprinted process; therefore, sandwich-like double-imprinted layers of ibuprofen could be constructed [66].

In another study, the time-dependent, phosphate-triggered release of ibuprofen was analyzed, and the loading capacity of ibuprofen in Zr-MOFs functionalized with amino groups was 55 mg/g for encapsulated ibuprofen [64]. By comparison, in the present study, the IBU loading capacity was 65.54 mg/g, mg IBU per g of carrier.

Dynamic dialysis is one of the most common methods for determining release kinetics from nanoparticle drug delivery systems [67]. The drug released from the nanocarriers first enters the solution inside the dialysis bag (donor compartment) and then permeates through the dialysis membrane to reach the bulk solution outside the dialysis bag (receiver compartment). Thus, the apparent drug release kinetics, which are measured by sampling the receiver compartment, are determined by both the actual drug release kinetics and drug permeation kinetics [68], and drug release takes place by osmosis.

In the Supplementary Material in Table S9, examples of UIO-66-type materials used for drug loading and release are presented.

### 3.3. Kinetic Modelling

Mathematical models are used to evaluate the kinetics and mechanisms of matrix-based drug release.

The model that best fit the release data was selected based on the coefficient of determination (*R*^2^) value calculated for each model. The model with the highest *R*^2^ value was considered the best fit for the release data. Nevertheless, none of these models can cover all possible release mechanisms.

The drug release mechanism of the Zr-MOFs was mainly diffusion-controlled, as plots of the amount released versus the square root of time were found to be linear when the Higuchi model was used. The coefficient of determination is approx. 0.97 for the linear fit of various formulations in the Higuchi model. When the log percentage of drug remaining to be released vs. time was plotted in accordance with the first-order equation, straight lines were obtained (*R*^2^ > 0.93), indicating that the drug release followed first-order kinetics as well, a model that is applicable for water-soluble drugs like captopril.

It is well known that the kinetics of drug release are usually explained by more than one mechanism. However, in the present case, the kinetic behavior of the system could be adequately described using the Higuchi model because drug release by diffusion proved to be the main mechanism. Drug release by carrier disintegration in a phosphate buffer was a secondary mechanism, which is a simple first-order kinetic model, which is in good agreement with the burst-like character of this release process because both drugs, captopril and ibuprofen, were loaded by adsorption [44,46,48,50]. These types of matrices with burst release are suitable for the treatment of acute infections or inflammation when an immediate high dose of drugs is required.

### 3.4. The Biosafety of Zr^*4*+^, Terephthalic Acid and the Resulted Zr-MOF

Zirconium is widespread in nature and used in biological systems. ZrOCl_2_·8H_2_O has low toxicity (LD_50_ [ZrOCl_2_·8H_2_O oral rate] = 2950 mg/kg), availability, low cost, moisture stability, ease of handling, recovery and reusability, safe potential catalytic activity, insensitivity to air, and good general stability [69,70]. Terephthalic acid is a free-flowing powder composed of round crystals. It forms needles if it recrystallizes slowly. The vapor pressure was low at 0.097 kPa at 250 °C, with sublimation at 402 °C and atmospheric pressure. Melting has been reported at 427 °C. Terephthalic acid is a stable, intractable compound with low solubility in most solvents such as water and alcohols. It is soluble in dimethyl formamide (DMF) and dimethyl sulfoxide (DMSO) [71]. Zr-based MOFs (Zr-MOFs), which exhibit rich structural characteristics, have excellent chemical, thermal, and water stability owing to their strong Zr-O bonds and stable secondary building units. Because zirconium is a biocompatible metal (the human body contains approximately 300 mg of zirconium, and the recommended daily ingestion of 4.15 mg/day), its applicability in zirconium-based MOFs and functionalized Zr-MOFs for biomedical applications and drug delivery fully demonstrates the biosafety criteria [5,7].

## 4. Materials and Methods

### 4.1. Synthesis

Zirconium oxychloride octahydrate (ZrOCl_2_·8H_2_O, 99%, Merck, Darmstadt, Germany), terephthalic acid (C_6_H_4_(CO_2_H)_2_, Aldrich, 98%, Steinheim, Germany); acetic acid glacial (CH_3_COOH, Chim reactiv S.R.L, 99.8%, Bucharest, Romania) N, N-dimethylformamide (DMF, 99%, Lach-Ner, Neratovice, Czech Republic), methanol (CH_3_OH, Riedel de Haën, 99.8%, Steinheim, Germany).

**Scheme 1:**
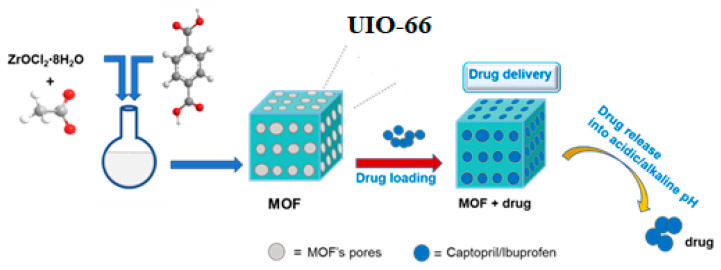
Schematic illustration of the chemical Zr-MOF synthesis and drug loading and release procedure.

Three Zr-MOF materials (**Zr-MOF1**, **Zr-MOF2** and **Zr-MOF3**, respectively) based on ZrOCl_2_·8H_2_O, 26 Eq acetic acid, and terephthalic acid in a DMF solution were synthesized and used for captopril and ibuprofen delivery (Scheme 1). The materials were obtained using the same amount and molar ratio of reactants under different reaction conditions or reactant addition order.

#### 4.1.1. Chemical Synthesis Procedure

**Zr-MOF1** material was synthetized according to our previously reported results [8]. In this case, the activation process was carried out using methanol, and a better result was obtained compared to activation in ethanol.

**Zr-MOF3**: ZrOCl_2_·8H_2_O (1.15 g, 3.5 mmol) and acetic acid (5.3 mL, 92 mmol) were separately sonicated for 10 min and the milky mixture was added to a DMF (18 mL) solution of terephthalic acid (0.4 g, 2.4 mmol). The reaction mixture was then stirred and heated at 120 °C for 24 h. White polycrystalline powder was collected by filtration and dried at room temperature. The powder was washed with 10 mL of DMF two times/day for three days and immersed in methanol, following the same procedure as washing in DMF. The solid was dried at 130 °C under vacuum for 12 h to yield the activated sample. The difference between **Zr-MOF1** and **Zr-MOF3** is the way of adding the reactants: in the case of **Zr-MOF1**, the starting reactants were added together from the start in the reaction media, and in the case of **Zr-MOF3**, ZrOCl_2_·8H_2_O was first added to acetic acid, terephthalic acid to DMF, and subsequently the obtained solutions were mixed.

#### 4.1.2. Solvothermal Method of Synthesis

**Zr-MOF2**: Terephthalic acid (0.4 g, 2.4 mmol), ZrOCl_2_·8H_2_O (1.15 g, 3.5 mmol) and acetic acid (5.3 mL) were dispersed in DMF (18 mL) in a Teflon liner. The mixture was stirred for 10 min, heated in an autoclave reactor at 120 °C for 24 h under high pressure, and slowly cooled to room temperature. The white polycrystalline powder was collected by filtration and air-dried. The sample was activated with methanol and dried at 130 °C under vacuum for 12 h.

### 4.2. Characterization

FT-IR spectra were recorded on a Cary 630 FT-IR spectrophotometer on KBr pellets in the range 4000–400 cm^−1^. The thermal stability of the Zr-MOFs was investigated using a thermogravimetric analyzer (TGA/SDTA 851/LF/1100 Mettler Toledo). The experiments were performed under a nitrogen flow of 50 mL/min in the temperature range of 25–800 °C (heating rate of 10 °C/min) and a dynamic atmosphere of air introduced at 800 °C, followed by a final isothermal heating for 15 min. The specific surface areas of the vacuum-dried Zr-MOF samples were measured by low-temperature nitrogen adsorption using a QuantaChrome Nova 1200e analyzer (Anton Paar GmbH, Graz, Austria). Before the measurements, the samples were outgassed at 130 °C for 12 h under vacuum. The surface area S_BET_ was determined using the Brunauer–Emmett–Teller (BET) method. Analysis of the pore size distribution was performed on the basis of nitrogen adsorption isotherms using the DFT method (cylindrical pore, NLDFT adsorption branch model). Small angle neutron scattering (SANS) measurements were performed using the Yellow Submarine SANS instrument [48] at the Budapest Neutron Centre. The **Zr-MOF2** and **Zr-MOF3** samples in powder form were loaded in 2 mm quartz cuvettes, and placed into the 7 mm diameter neutron beam. The used instrument set-up parameters were: sample to detector distances: 1.3 m and 5.5 m, wavelength: 4.2 Å and 12.1 Å. The absorbance measurements for captopril (at 209 nm) and ibuprofen (at 264 nm) were determined using a Cary 60 spectrophotometer instrument. X-ray diffraction was performed on a Bruker AXS D8 Discover diffractometer equipped with a Göbel mirror and scintillation detector using Cu Kα radiation. Measurements were performed over an interval of 5–40° with a step size of 0.02° and scan speed of 0.25°/min. Scanning Electron Microscopy (SEM) and Energy Dispersive X-ray Spectrometry (EDX) investigations were performed on a Zeiss LEO 1540XB dual-beam system equipped with an Oxford UltimMax 40 Si drift detector EDX. A 5 keV beam energy was applied, and the dead time was less than 50%.

### 4.3. Drug Loading and Release Procedures

#### 4.3.1. Chemicals for Captopril Loading and Release Procedure

Pure captopril ((2S)-1-[(2S)-2-methyl-3-sulfanylpropanoyl] pyrrolidine-2-carboxylic acid), provided free of charge by a pharmaceutical company); hydrochloric acid (HCl 37%, SC Silal Trading SRL, Bucharest, Romania); natrium chloride (NaCl, SC Chimopar Trading SRL); disodium hydrogen phosphate (Na_2_HPO_4_·12H_2_O, Reactivul Bucuresti, Bucharest, Romania); monosodium phosphate (NaH_2_PO_4_·H_2_O, Reactivul Bucuresti, Bucharest, Romania).

#### 4.3.2. Captopril Loaded by Adsorption

A 0.1 M captopril solution was prepared using NaCl 0.9%. A classic procedure for loading was used by soaking 0.2 g of **Zr-MOF1** material with 10 mL of 0.1 M captopril solution (containing 200 mg of captopril prepared in 0.9% NaCl, with continuous stirring at room temperature). After 24 h, **Zr-MOF1-CP** (the drug-loaded **Zr-MOF1** carrier) was filtered and left to dry at room temperature for another day. The filtrates were tested spectrophotometrically at 209 nm for any remaining free drug. The filtrate was measured (cca 7 mL for each experiment) to determine the concentration of captopril that was not entrapped in the matrix. The solid was then used for in vitro drug release.

#### 4.3.3. In Vitro Captopril Release Procedures

To mimic in vitro pH conditions from the stomach and intestine where the drugs finally reach, drug release tests were performed in pH 1.2 hydrochloric acid solution and in pH 7.4 phosphate-buffered solution, respectively.

The drug-loaded carrier was soaked in a beaker filled with 200 mL solutions of different pH values. The experiments were performed at room temperature under stirring, to mimic the peristaltic motility motion of human organisms in vitro [45].

Next, periodically (every 5 min in the first 130 min; every 30 min in the next 4 h and the next day, after 23.25 h), samples of 3 mL of buffer were removed for analysis and replaced with another 3 mL of fresh buffer solution of different pH values.

The 3 mL samples were filtered before spectrophotometric analyses. In some cases, if the samples were too concentrated in the drug, they were diluted to be measurable by spectrophotometry and the concentrations were calculated accordingly.

#### 4.3.4. Captopril Release Procedure Using the Dialysis Membrane (Regenerate Cellulose Tubular Membrane Zellu Trans MWCO: 12,000–14,000; Pore Size = 25 Å; Wall Thickness = 40 µm; Karlsruhe, Germany)

**Zr-MOF3** was used for the captopril loading by adsorption using the same procedure as that described above for the **Zr-MOF1** material. The **Zr-MOF3-CP** material containing captopril obtained. Using the **Zr-MOF3-CP** material, the in vitro captopril release procedure was repeated, as described above for the **Zr-MOF1-CP** material, except for the following: the drug-containing carrier (**Zr-MOF3-CP** material) was first introduced into a dialysis membrane forming a bag closed at both ends and subsequently soaked inhydrochloric pH 1.2 acidic buffer; the next day, the same dialysis membrane-containing carrier (assuming that, overnight, by diffusion all the drug resorb into the matrix) was soaked in the phosphate-buffered solution and the release experiments were continued as described above for the first material. This experiment was conducted in order to mimic how the drug-containing carrier travels from stomachal media to intestinal media.

#### 4.3.5. Chemicals for Ibuprofen Loading and Release Procedure

Pure ibuprofen (2-[4-(2-methylpropyl)phenyl]propanoic acid), provided free of charge by a pharmaceutical company), sodium chloride (NaCl, SC Chimopar Trading SRL), disodium hydrogen phosphate (Na_2_HPO_4_·12H_2_O, Reactivul Bucuresti), monosodium phosphate (NaH_2_PO_4_·H_2_O, Reactivul Bucuresti).

#### 4.3.6. Ibuprofen Loaded by Adsorption

An ibuprofen solution (20 mL) was prepared by dissolving 55 mg ibuprofen in 10 mL of 0.9% NaCl solution, followed by the addition of 10 mL of ethanol. A classic procedure for loading was used by soaking 0.8111 g of **Zr-MOF2** material in 20 mL of the obtained ibuprofen solution while stirring for 24 h. The next day, **Zr-MOF2-IBU** (the drug-loaded **Zr-MOF2** carrier) was filtered and left to dry at room temperature for another day. The filtrate was tested spectrophotometrically for free drug remnants. The filtrate (16 mL) was measured to determine the concentration of ibuprofen that was not entrapped in the matrix. The solid was used for in vitro drug release.

#### 4.3.7. In Vitro Ibuprofen Release Procedures

To mimic the in vitro pH conditions from the intestine where the drugs finally reach, drug release tests were performed in a phosphate-buffered solution of pH 7.4.

The drug-loaded carrier was soaked in a beaker filled with 200 mL of the buffer solution. The experiments were performed at room temperature under stirring to mimic in vitro intestinal peristaltic motility [45].

Next, periodically (every 5 min in the first 160 min and every 30 min in the next hours), samples of 3 mL of buffer were removed for analysis and replaced with another 3 mL of fresh buffer solution.

The 3 mL samples were then filtered before the spectrophotometric analyses, and they were measured using a spectrophotometer at 264 nm. To calculate the ibuprofen concentration, a molar extinction coefficient having the value of 365.6 L/mol was used.

#### 4.3.8. Solutions Prepared for the Drug Loading and Release Experiments

pH 7.4 sodium phosphate-buffered solution (Preparation: A 50 mL solution of 0.2 M of Na_2_HPO_4_·12H_2_O was prepared by dissolving 3.58 g in 50 mL distilled water (solution A), a 50 mL solution of 0.2 M (solution B) of NaH_2_PO_4_·H_2_O was prepared by dissolving 1.38 g in 50 mL of distilled water (solution B); after that, 40.5 mL solution A was added to solution B (9.5 mL) in order to reach a final volume of 100 mL of distilled water. Phosphate-buffered solution (200 mL) was used for each drug-release experiment.

Different batches of solutions (concentration 0.1M and pH 1.2) were prepared by dissolving 4.4 mL of hydrochloric acid (with a concentration of 37%) in distilled water to reach a final volume of 500 mL, resulting in a solution with pH 1.2.

The 0.1 M captopril stock solution was prepared in 0.9% NaCl solution.

Next, successive dilutions of the stock solution were performed in 0.9% NaCl and solutions with different pH value to set different calibration curves. The final solutions were measured at a wavelength of 209 nm.

## 5. Conclusions

The purpose of this study was to develop controlled drug-delivery systems loaded with water-soluble and reduced water-soluble drugs, intended to be evaluated for in vitro tests for oral administration, to improve the dissolution rate and ensure controlled drug release. The PXRD patterns of the carriers were characterized by similar features that point to well-crystallized phases specific to the UIO-66-type materials demonstrating as well by SEM that the materials are composed of small and agglomerate crystals. Thermogravimetric analysis demonstrated that both materials had approximately two linker deficiencies per Zr_6_ formula unit. Pores with an average dimension of ~4 nm were revealed by nitrogen porosimetry, and the average sizes of the Zr-MOF nanocrystals were suggested to be approximately 30 nm, as revealed by SANS. The specific surface area, total pore volume, and pore diameter of the drug delivery system are the main factors responsible for improving the maximum drug-loading capacity. The test results confirmed that the drug carrier systems allowed for a very high drug loading of approximately 99.78% in weight. Thus far, these materials have shown good applicability as drug adsorbents in polluted waters. In vitro drug release testing demonstrated that the Zr-MOF carrier showed slower release rates in an acidic medium than in a phosphate buffer. The rate of drug release from the Zr-MOF materials was dependent on the pore size, but there was no difference between the samples with approximately the same pore size. The low cumulative drug release was realized in a burst-like process owing to the rapid establishment of dynamic equilibrium between the dissolved and bound forms of the drug. The drug release mechanism in various pH media, including a phosphate buffer, for all samples obeyed Fickian diffusion. The drug release was followed by carrier disintegration. The obtained Zr-MOF materials were more favorable for the adsorption and controlled release of water-soluble drugs than the less soluble drug. Thus, the effect of structural properties on controlled captopril release efficiency was also tested. This knowledge facilitates tailoring the pore network for specific usage in biological/medical applications as suitable matrices for efficient encapsulation of drugs and their ability to provide controlled release. We plan to obtain functionalized materials with different groups, such as aminopropyl, to improve the release rate and test the drug release over a period of more than 48 h, which opens the opportunity for considering formulations from only one daily administration to one per two days administration for topical release. Different captopril release behaviors were observed when the experiments were performed using a permeable dialysis membrane as an osmotically controlled release system.

## Figures and Tables

**Table 1:** Textural parameter for the obtained MOFs.

Sample	Surface Area, BET, m^2^/g	Pore Size Distribution, BJH Ads, nm	Pore Size Distribution BJH Des, nm	Pore Width, DFT, nm	Average Pore Size, nm	Total Pore Volume, cm^3^/g	Micropore Surface Area, m^2^/g	Total Micropore Volume, cm^3^/g
**Zr-MOF1**	1123	3.09	24.36	1.77	3.78	1.06	904	0.38
**Zr-MOF2**	912	3.47	3.28	2.35	4.04	0.92	678	0.29
**Zr-MOF3**	731	3.41	12.50	2.19	3.91	0.71	482	0.20

**Table 2:** Loading efficiency and loading capacity of drugs calculated for each experimental procedure.

Drug Loading				Drug Release		
Drug Used for Entrapment	Drug Effectively Entrapped	Loading Efficiency (%)	Loading Capacity (mg Drug/g of Carrier)	Calibration Curve	Release Buffer	Cumulative Drug Release
**Captopril**						
Captopril 200 mg	199.56 mg	99.78	997.8	HCl buffer pH = 1.2	HCl buffer pH = 1.2	15.9% (in 23.5 h)
Captopril 200 mg	199.612 mg	99.8	998.08	NaCl 0.9%	Phosphate Buffer pH = 7.4	31.38% (in 23.5 h)
**Ibuprofen**						
55 mg	53.1565 mg	96.65	65.46	NaCl 0.9%	Phosphate Buffer pH = 7.4	65.62% (in 6.17 h)
**Captopril (calculated for the dialysis experiment)**						
199.9 mg	198.9198 mg	99.51	994.6	HCl buffer pH = 1.2	HCl buffer pH = 1.2	99.51% (in 1.5 h)
				NaCl 0.9%	Phosphate Buffer pH = 7.4	75.29% (in 23.5 h)

**Table 3:** The % of captopril release after 5.6 h and after 23.25 h in different buffer solutions.

Sample	Time of Release (h)	% of Captopril Release	
		pH = 1.2	pH = 7.4
**Zr-MOF1-CP**	5.6 h	15.72	30.08
**Zr-MOF1-CP**	23.25 h	15.94	31.38

**Table 4:** The % of ibuprofen release after 5.6 h and 6.17 h.

Sample	Time of Release (h)	% of Ibuprofen Release pH = 7.4
**Zr-MOF2-IBU**	5.6 h	64.54
**Zr-MOF2-IBU**	6.17 h	65.62

**Table 5:** The % of captopril release after 1.5 h and after 5.6 h in different buffer solutions using the dialysis membrane.

Sample	Time of Release (h)	% of Captopril Release	
		pH = 1.2	pH = 7.4
**Zr-MOF3-CP**	1.5 h	99.51	-
**Zr-MOF3-CP**	5.6 h	-	75.29

## Data Availability

Not applicable.

## Supplementary Materials

The following supporting information can be downloaded at: https://www.mdpi.com/article/10.3390/ijms241813887/s1. References [72,73,74,75,76,77,78,79] are cited in Supplementary Material.
