# Supplementary material for: Performance of Zr-Based Metal–Organic Framework Materials as In Vitro Systems for the Oral Delivery of Captopril and Ibuprofen
Source: Int J Mol Sci. 2023 Sep 9;24(18):13887. doi: 10.3390/ijms241813887 (PMC10531200; doi:10.3390/ijms241813887)
Supplement: Supplementary file 1 [file ijms-24-13887-s001.zip › ijms-2538483-supplementary.pdf]

International Journal of *Molecular Sciences*

MDPI

Suplementary Material

The performances of Zr- based metal-organic frameworks materials as In vitro Systems for the Oral Delivery of Captopril and Ibuprofen

Carmen Cretu [1], Roxana Nicola[1], Sorin-Alin Marinescu [1], Elena-Mirela Picioruș [1] , Mariana Suba [1], Corina Duda-Seiman[2], Adel Len [3,4], Levente Illés [5], Zsolt Endre Horváth [5] and Ana-Maria Putz [1,*]

[1] "Coriolan Drăgulescu" Institute of Chemistry, Bv. Mihai Viteazul, No. 24, 300223 Timisoara, Romania; carmencretu78@gmail.com; cc.roxana@yahoo.com; sorin.alin.marinescu@gmail.com; e_piciorus@yahoo.com; marianasuba@gmail.com; putzanamaria@acad-icht.tm.edu.ro;

[2] West University of Timisoara, Biology-Chemistry Department, Johann Heinrich Pestalozzi no. 16, 300115; corina.seiman78@e-uvt.ro

[3] Institute for Energy Security and Environmental Safety, Centre for Energy Research, Konkoly-Thege Miklós út 29-33, 1121 Budapest, Hungary, adel.len@ek-cer.hu

[4] Faculty of Engineering and Information Technology, University of Pécs, Boszorkány street 2, 7624 Pécs, Hungary.

[5] Institute for Technical Physics and Material Science, Centre for Energy Research, Konkoly-Thege út 29-33, 1121 Budapest, Hungary; horvath.zsolt.endre@ek-cer.mta.hu, illes.levente@ek-cer.hu

a

* Correspondence: putzanamaria@acad-icht.tm.edu.ro

Kintetics for the first hours of release

Table S1: The calculated parameter for the release kinetic of captopril in acidic buffer

**Time (Hr)**	**cumulative % drug released**	**% drug remaining**	**Square root time**	**log Cumu % drug remaining**	**log time**	**log Cumu % drug released**	**% Drug released**	**Cube Root of % drug Remaining(Wt)**	**Wo-Wt**	**Cube Root of % drug (Wo)**
**0.00**	0.00	**100.00**	**0.00**	**2.00**	**0.00**	**0.00**	**0.00**	**4.64**	**0.00**	
**0.08**	0.57	99.43	0.29	2.00	-1.08	-0.24	0.57	4.63	0.01	4.64
**0.17**	0.86	99.14	0.41	2.00	-0.78	-0.06	0.29	4.63	0.01	
**0.25**	1.00	99.00	0.50	2.00	-0.60	0.00	0.14	4.63	0.02	
**0.33**	1.43	98.57	0.58	1.99	-0.48	0.16	0.43	4.62	0.02	
**0.42**	1.71	98.29	0.65	1.99	-0.38	0.23	0.27	4.62	0.03	
**0.50**	2.07	97.93	0.71	1.99	-0.30	0.32	0.36	4.61	0.03	
**0.58**	3.07	96.93	0.76	1.99	-0.23	0.49	1.00	4.59	0.05	
**0.67**	4.21	95.79	0.82	1.98	-0.18	0.62	1.14	4.58	0.07	
**0.75**	5.72	94.28	0.87	1.97	-0.12	0.76	1.52	4.55	0.09	
**0.83**	6.02	93.98	0.91	1.97	-0.08	0.78	0.30	4.55	0.10	
**0.92**	6.08	93.92	0.96	1.97	-0.04	0.78	0.06	4.55	0.10	
**1.00**	6.32	93.68	1.00	1.97	0.00	0.80	0.24	4.54	0.10	
**1.08**	6.41	93.59	1.04	1.97	0.03	0.81	0.09	4.54	0.10	
**1.17**	6.45	93.55	1.08	1.97	0.07	0.81	0.04	4.54	0.10	
**1.25**	6.66	93.34	1.12	1.97	0.10	0.82	0.22	4.54	0.11	
**1.33**	6.83	93.17	1.15	1.97	0.12	0.83	0.16	4.53	0.11	
**1.42**	7.30	92.70	1.19	1.97	0.15	0.86	0.47	4.53	0.12	
**1.50**	7.54	92.46	1.22	1.97	0.18	0.88	0.25	4.52	0.12	
**1.58**	7.85	92.15	1.26	1.96	0.20	0.89	0.30	4.52	0.13	
**1.67**	8.79	91.21	1.29	1.96	0.22	0.94	0.95	4.50	0.14	
**1.75**	8.95	91.05	1.32	1.96	0.24	0.95	0.16	4.50	0.14	
**1.83**	9.04	90.96	1.35	1.96	0.26	0.96	0.09	4.50	0.14	
**1.92**	9.14	90.86	1.38	1.96	0.28	0.96	0.10	4.50	0.15	
**2.00**	11.41	88.59	1.41	1.95	0.30	1.06	2.27	4.46	0.18	
**2.08**	11.64	88.36	1.44	1.95	0.32	1.07	0.23	4.45	0.19	
**2.17**	12.01	87.99	1.47	1.94	0.34	1.08	0.38	4.45	0.19	
**2.67**	12.32	87.68	1.63	1.94	0.43	1.09	0.31	4.44	0.20	
**3.17**	13.25	86.75	1.78	1.94	0.50	1.12	0.93	4.43	0.22	
**3.67**	13.68	86.32	1.91	1.94	0.56	1.14	0.43	4.42	0.22	
**4.17**	13.89	86.11	2.04	1.94	0.62	1.14	0.21	4.42	0.23	
**4.67**	14.59	85.41	2.16	1.93	0.67	1.16	0.69	4.40	0.24	
**5.17**	14.92	85.08	2.27	1.93	0.71	1.17	0.34	4.40	0.24	
**5.67**	15.72	84.28	2.38	1.93	0.75	1.20	0.80	4.38	0.26	
**23.25**	15.94	84.06	4.82	1.92	1.37	1.20	0.22	4.38	0.26	

Table S2: The calculated parameter for the release kinetic of captopril in phosphate buffer

ime (Hr)	cumulative % drug released	% drug remaining	Square root time	log Cumulative % drug remainining	log time	log Cumu % drug released	% Drug released	Cube Root of % drug Remaining(Wt)	Wo-Wt
0.00	**0.00**	**100.00**	**0.00**	**2.00**	**0.00**	**0.00**	**0.00**	**4.64**	**0.00**
0.08	1.25	98.75	0.29	1.99	-1.08	0.10	1.25	4.62	0.02
0.17	1.96	98.04	0.41	1.99	-0.78	0.29	0.70	4.61	0.03
0.25	2.70	97.30	0.50	1.99	-0.60	0.43	0.74	4.60	0.04
0.33	3.50	96.50	0.58	1.98	-0.48	0.54	0.80	4.59	0.06
0.42	4.55	95.45	0.65	1.98	-0.38	0.66	1.05	4.57	0.07
0.50	5.17	94.83	0.71	1.98	-0.30	0.71	0.62	4.56	0.08
0.58	6.22	93.78	0.76	1.97	-0.23	0.79	1.05	4.54	0.10
0.67	7.51	92.49	0.82	1.97	-0.18	0.88	1.29	4.52	0.12
0.75	8.42	91.58	0.87	1.96	-0.12	0.93	0.91	4.51	0.13
0.83	9.34	90.66	0.91	1.96	-0.08	0.97	0.92	4.49	0.15
0.92	10.38	89.62	0.96	1.95	-0.04	1.02	1.05	4.48	0.17
1.00	10.88	89.12	1.00	1.95	0.00	1.04	0.50	4.47	0.18
1.08	11.62	88.38	1.04	1.95	0.03	1.07	0.74	4.45	0.19
1.17	12.20	87.80	1.08	1.94	0.07	1.09	0.58	4.44	0.20
1.25	13.13	86.87	1.12	1.94	0.10	1.12	0.93	4.43	0.21
1.33	13.68	86.32	1.15	1.94	0.12	1.14	0.55	4.42	0.22
1.42	14.50	85.50	1.19	1.93	0.15	1.16	0.82	4.41	0.24
1.50	15.56	84.44	1.22	1.93	0.18	1.19	1.06	4.39	0.25
1.58	16.85	83.15	1.26	1.92	0.20	1.23	1.29	4.36	0.28
1.67	17.53	82.47	1.29	1.92	0.22	1.24	0.68	4.35	0.29
1.75	18.45	81.55	1.32	1.91	0.24	1.27	0.92	4.34	0.31
1.83	19.75	80.25	1.35	1.90	0.26	1.30	1.30	4.31	0.33
1.92	20.33	79.67	1.38	1.90	0.28	1.31	0.58	4.30	0.34
2.00	21.40	78.60	1.41	1.90	0.30	1.33	1.06	4.28	0.36
2.08	22.70	77.30	1.44	1.89	0.32	1.36	1.30	4.26	0.38
2.17	23.52	76.48	1.47	1.88	0.34	1.37	0.82	4.24	0.40
2.67	24.45	75.55	1.63	1.88	0.43	1.39	0.93	4.23	0.41
3.17	25.52	74.48	1.78	1.87	0.50	1.41	1.07	4.21	0.43
3.67	26.45	73.55	1.91	1.87	0.56	1.42	0.93	4.19	0.45
4.17	27.04	72.96	2.04	1.86	0.62	1.43	0.59	4.18	0.46
4.67	27.72	72.28	2.16	1.86	0.67	1.44	0.68	4.17	0.48
5.17	29.01	70.99	2.27	1.85	0.71	1.46	1.29	4.14	0.50
5.67	30.08	69.92	2.38	1.84	0.75	1.48	1.07	4.12	0.52

23.25	31.38	68.62	4.82	1.84	1.37	1.50	1.30	4.09	0.55

Table S3: The calculated parameter for the release kinetic of ibuprofen in phosphate buffer

Time (Hr)	cumulative % drug released	% drug remaining	Square root time	log Cumu % drug remainining	log time	log Cumu % drug released	% Drug released	Cube Root of % drug Remaining(Wt)	Wo-Wt
**0.00**	0.00	100.00	0.00	2.00	0.00	0.00	0.00	4.64	0.00
**0.08**	3.40	96.60	0.29	1.98	-1.08	0.53	3.40	4.59	0.05
**0.17**	6.51	93.49	0.41	1.97	-0.78	0.81	3.10	4.54	0.10
**0.25**	9.48	90.52	0.50	1.96	-0.60	0.98	2.98	4.49	0.15
**0.33**	12.75	87.25	0.58	1.94	-0.48	1.11	3.27	4.44	0.21
**0.42**	15.83	84.17	0.65	1.93	-0.38	1.20	3.08	4.38	0.26
**0.50**	18.56	81.44	0.71	1.91	-0.30	1.27	2.73	4.33	0.31
**0.58**	21.15	78.85	0.76	1.90	-0.23	1.33	2.58	4.29	0.35
**0.67**	23.64	76.36	0.82	1.88	-0.18	1.37	2.50	4.24	0.40
**0.75**	26.03	73.97	0.87	1.87	-0.12	1.42	2.39	4.20	0.44
**0.83**	28.47	71.53	0.91	1.85	-0.08	1.45	2.44	4.15	0.49
**0.92**	30.72	69.28	0.96	1.84	-0.04	1.49	2.25	4.11	0.53
**1.00**	32.90	67.10	1.00	1.83	0.00	1.52	2.18	4.06	0.58
**1.08**	35.02	64.98	1.04	1.81	0.03	1.54	2.12	4.02	0.62
**1.17**	36.98	63.02	1.08	1.80	0.07	1.57	1.96	3.98	0.66
**1.25**	38.95	61.05	1.12	1.79	0.10	1.59	1.96	3.94	0.70
**1.33**	40.74	59.26	1.15	1.77	0.12	1.61	1.79	3.90	0.74
**1.42**	42.49	57.51	1.19	1.76	0.15	1.63	1.75	3.86	0.78
**1.50**	44.39	55.61	1.22	1.75	0.18	1.65	1.90	3.82	0.83
**1.58**	46.21	53.79	1.26	1.73	0.20	1.66	1.82	3.77	0.87
**1.67**	48.00	52.00	1.29	1.72	0.22	1.68	1.79	3.73	0.91
**1.75**	49.57	50.43	1.32	1.70	0.24	1.70	1.57	3.69	0.95
**1.83**	51.10	48.90	1.35	1.69	0.26	1.71	1.52	3.66	0.99
**1.92**	52.54	47.46	1.38	1.68	0.28	1.72	1.45	3.62	1.02
**2.00**	53.96	46.04	1.41	1.66	0.30	1.73	1.42	3.58	1.06
**2.08**	55.27	44.73	1.44	1.65	0.32	1.74	1.30	3.55	1.09
**2.17**	56.49	43.51	1.47	1.64	0.34	1.75	1.23	3.52	1.12
**2.67**	57.76	42.24	1.63	1.63	0.43	1.76	1.27	3.48	1.16
**3.17**	58.99	41.01	1.78	1.61	0.50	1.77	1.23	3.45	1.19
**3.67**	60.18	39.82	1.91	1.60	0.56	1.78	1.19	3.41	1.23
**4.17**	61.27	38.73	2.04	1.59	0.62	1.79	1.09	3.38	1.26
**4.67**	62.40	37.60	2.16	1.58	0.67	1.80	1.13	3.35	1.29

**5.17**	63.41	36.59	2.27	1.56	0.71	1.80	1.01	3.32	1.32
**5.67**	64.54	35.46	2.38	1.55	0.75	1.81	1.13	3.29	1.36
**6.17**	65.62	34.38	2.48	1.54	0.79	1.82	1.08	3.25	1.39

Table S4: The calculated parameter for the release kinetic of captopril in acidic buffer using a dialysis membrane

Time (Hr)	cumulative % drug released	% drug remaining	Square root time	log Cumu % drug remaining	log time	log Cumu % drug released	% Drug released	Cube Root of % drug Remaining (Wt)	Wo-Wt	Cube Root of % drug (Wo)
0.00	0.00	100.00	0.00	2.00	0.00	0.00	0.00	4.64	0.00	
0.08	5.54	94.46	0.29	1.98	-1.08	0.74	5.54	4.55	0.09	4.64
0.17	11.00	89.00	0.41	1.95	-0.78	1.04	5.46	4.46	0.18	
0.25	16.67	83.33	0.50	1.92	-0.60	1.22	5.66	4.37	0.27	
0.33	22.07	77.93	0.58	1.89	-0.48	1.34	5.40	4.27	0.37	
0.42	28.52	71.48	0.65	1.85	-0.38	1.46	6.45	4.15	0.49	
0.50	34.69	65.31	0.71	1.81	-0.30	1.54	6.17	4.03	0.61	
0.58	40.37	59.63	0.76	1.78	-0.23	1.61	5.68	3.91	0.74	
0.67	45.57	54.43	0.82	1.74	-0.18	1.66	5.20	3.79	0.85	
0.75	50.72	49.28	0.87	1.69	-0.12	1.71	5.15	3.67	0.98	
0.83	55.91	44.09	0.91	1.64	-0.08	1.75	5.19	3.53	1.11	
0.92	61.22	38.78	0.96	1.59	-0.04	1.79	5.32	3.38	1.26	
1.00	66.81	33.19	1.00	1.52	0.00	1.82	5.58	3.21	1.43	
1.08	72.28	27.72	1.04	1.44	0.03	1.86	5.47	3.03	1.62	
1.17	77.84	22.16	1.08	1.35	0.07	1.89	5.56	2.81	1.83	
1.25	83.66	16.34	1.12	1.21	0.10	1.92	5.82	2.54	2.10	
1.33	88.79	11.21	1.15	1.05	0.12	1.95	5.13	2.24	2.40	
1.42	94.19	5.81	1.19	0.76	0.15	1.97	5.40	1.80	2.84	
1.50	99.51	0.49	1.22	-0.31	0.18	2.00	5.32	0.79	3.85	

Table S5: The calculated parameter for the release kinetic of captopril in phosphate buffer using dialysis membrane

Time (Hr)	cumulative % drug released	% drug remaining	Square root time	log Cumulative % drug remainining	log time	log Cumu % drug released	% Drug released	Cube Root of % drug Remaining(Wt)	Wo-Wt	Cube Root of % drug (Wo)
0.00	0.00	100.00	0.00	2.00	0.00	0.00	0.00	4.64	0.00	
0.08	2.18	97.82	0.29	1.99	-1.08	0.34	2.18	4.61	0.03	4.64
0.17	4.28	95.72	0.41	1.98	-0.78	0.63	2.10	4.57	0.07	
0.25	6.41	93.59	0.50	1.97	-0.60	0.81	2.13	4.54	0.10	
0.33	8.48	91.52	0.58	1.96	-0.48	0.93	2.07	4.51	0.14	
0.42	10.68	89.32	0.65	1.95	-0.38	1.03	2.20	4.47	0.17	
0.50	12.93	87.07	0.71	1.94	-0.30	1.11	2.25	4.43	0.21	
0.58	15.08	84.92	0.76	1.93	-0.23	1.18	2.16	4.40	0.25	
0.67	17.32	82.68	0.82	1.92	-0.18	1.24	2.24	4.36	0.29	
0.75	19.51	80.49	0.87	1.91	-0.12	1.29	2.18	4.32	0.32	
0.83	21.63	78.37	0.91	1.89	-0.08	1.34	2.13	4.28	0.36	
0.92	23.64	76.36	0.96	1.88	-0.04	1.37	2.01	4.24	0.40	
1.00	25.70	74.30	1.00	1.87	0.00	1.41	2.06	4.20	0.44	
1.08	27.88	72.12	1.04	1.86	0.03	1.45	2.18	4.16	0.48	
1.17	30.09	69.91	1.08	1.84	0.07	1.48	2.21	4.12	0.52	
1.25	32.38	67.62	1.12	1.83	0.10	1.51	2.29	4.07	0.57	
1.33	34.65	65.35	1.15	1.82	0.12	1.54	2.26	4.03	0.61	
1.42	36.68	63.32	1.19	1.80	0.15	1.56	2.03	3.99	0.66	
1.50	38.85	61.15	1.22	1.79	0.18	1.59	2.17	3.94	0.70	
1.58	41.15	58.85	1.26	1.77	0.20	1.61	2.30	3.89	0.75	
1.67	43.17	56.83	1.29	1.75	0.22	1.64	2.03	3.84	0.80	
1.75	45.53	54.47	1.32	1.74	0.24	1.66	2.35	3.79	0.85	
1.83	48.09	51.91	1.35	1.72	0.26	1.68	2.56	3.73	0.91	
1.92	50.84	49.16	1.38	1.69	0.28	1.71	2.74	3.66	0.98	
2.00	53.17	46.83	1.41	1.67	0.30	1.73	2.33	3.60	1.04	
2.08	55.73	44.27	1.44	1.65	0.32	1.75	2.56	3.54	1.10	
2.17	58.29	41.71	1.47	1.62	0.34	1.77	2.56	3.47	1.17	
2.67	60.85	39.15	1.63	1.59	0.43	1.78	2.56	3.40	1.25	
3.17	63.36	36.64	1.78	1.56	0.50	1.80	2.51	3.32	1.32	
3.67	65.81	34.19	1.91	1.53	0.56	1.82	2.45	3.25	1.40	
4.17	68.27	31.73	2.04	1.50	0.62	1.83	2.46	3.17	1.48	
4.67	70.67	29.33	2.16	1.47	0.67	1.85	2.40	3.08	1.56	
5.17	73.00	27.00	2.27	1.43	0.71	1.86	2.33	3.00	1.64	
5.67	75.29	24.71	2.38	1.39	0.75	1.88	2.30	2.91	1.73	
23.25	31.38	68.62	4.82	1.84	1.37	1.50	-43.91	4.09	0.55	

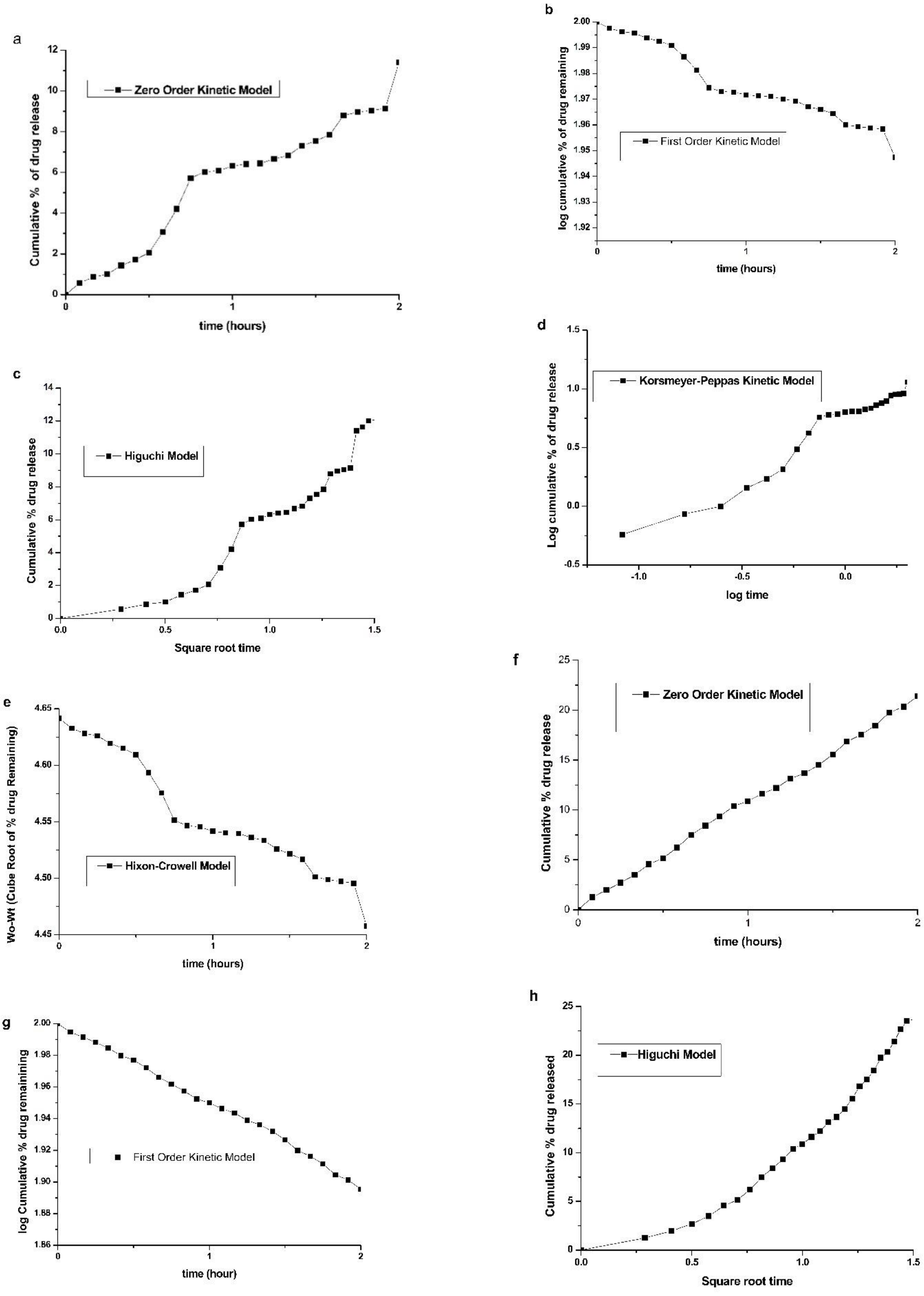
a
Zero Order Kinetic Model
Cumulative % of drug release
time (hours)
b
First Order Kinetic Model
log cumulative % of drug remaining
time (hours)
c
Higuchi Model
Cumulative % drug release
Square root time
d
Korsmeyer-Peppas Kinetic Model
Log cumulative % of drug release
log time
e
Hixon-Crowell Model
Wo-Wt (Cube Root of % drug Remaining)
time (hours)
f
Zero Order Kinetic Model
Cumulative % drug release
time (hours)
g
First Order Kinetic Model
log Cumulative % drug remaining
time (hour)
h
Higuchi Model
Cumulative % drug released
Square root time

i

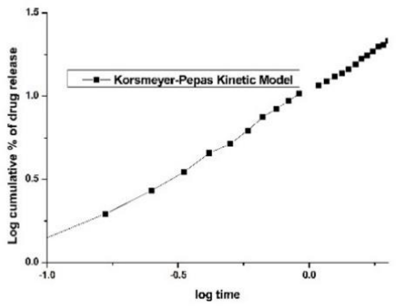

j

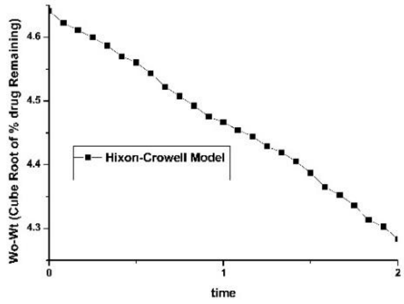

Figure S1. Different kinetic models applied for the captopril release from **Zr-MOF1-CP** in different buffers, of pH=1.2 (a,b,c,d,e) and of pH=7.4 (f,g,h,i,j). Straight solid lines are kinetics fitted to the first 2-hour data.

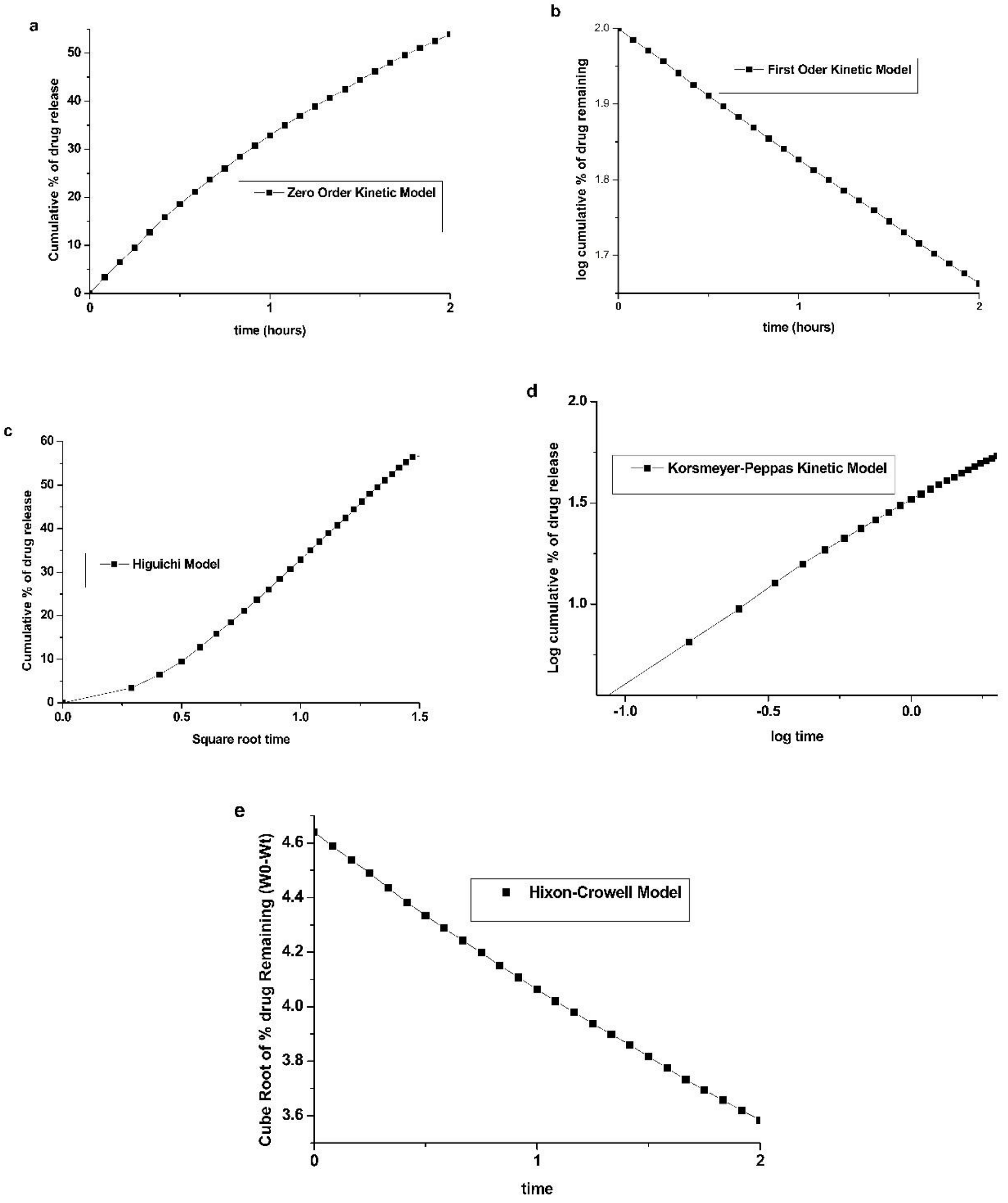

Figure S2. Different kinetic models applied for the ibuprofen release from **Zr-MOF2-IBU** in buffer of pH=7.4 (a-e). Straight solid lines are kinetics fitted to the first 2-hour data.

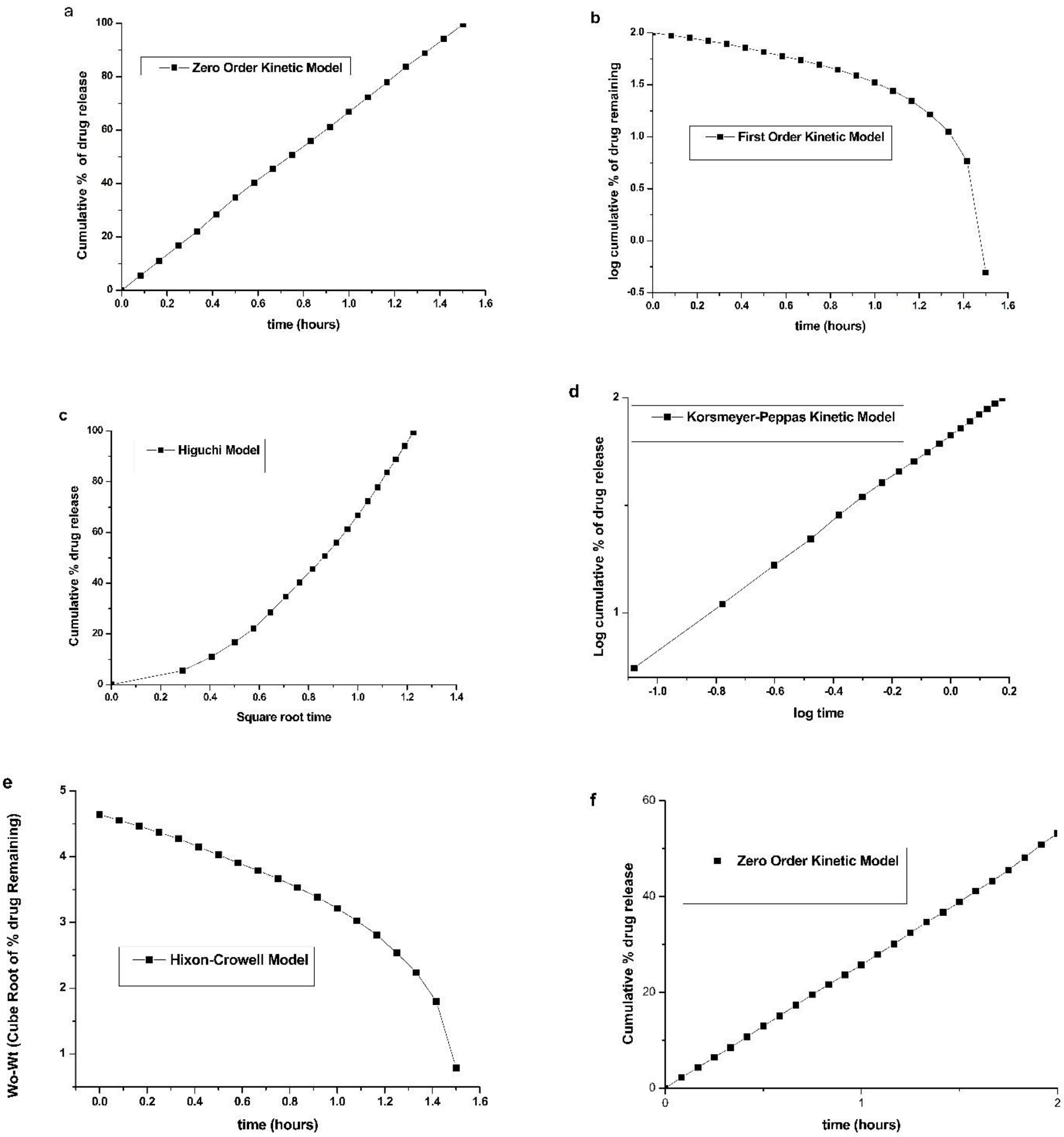
a
Zero Order Kinetic Model
Cumulative % of drug release
time (hours)
b
First Order Kinetic Model
log cumulative % of drug remaining
time (hours)
c
Higuchi Model
Cumulative % drug release
Square root time
d
Korsmeyer-Peppas Kinetic Model
Log cumulative % of drug release
log time
e
Hixon-Crowell Model
Wo-Wt (Cube Root of % drug Remaining)
time (hours)
f
Zero Order Kinetic Model
Cumulative % drug release
time (hours)

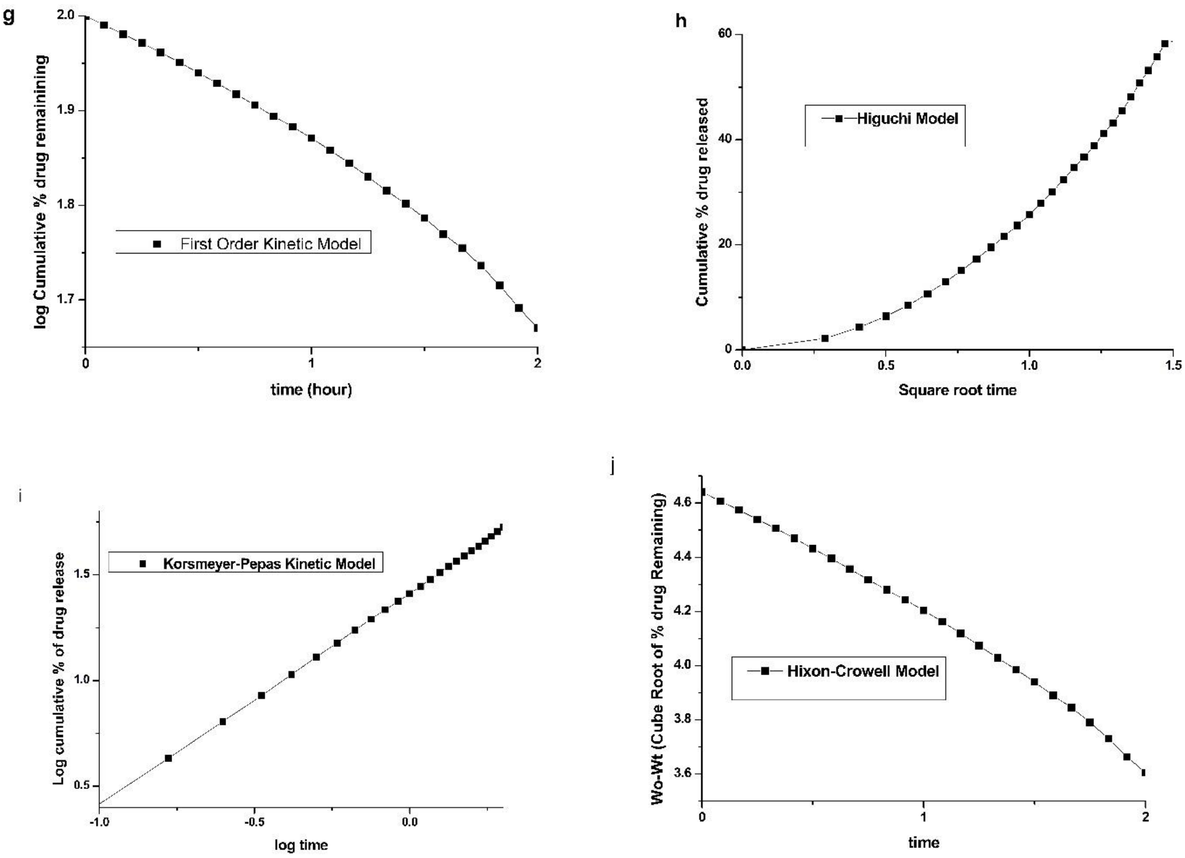

Figure S3. Different kinetic models applied for the captopril release from **Zr-MOF3-CP** in different buffers, of pH=1.2 (a,b,c,d,e) applied only for the first 1.5 hours of release and of pH=7.4 (f,g,h,i,j) applied for the first 2 hours of release by using a dialysis membrane. Straight solid lines are kinetics fitted.

Table S6. The calculated coefficient of determination (R^2) for the release kinetic in acidic or phosphate buffer for captopril from **Zr-MOF1-CP.**

Kinetic model	Zero Order	First Order	Higuchi	Korsmeyer-Peppas	Hixson–Crowell
**Applied for the first 5.66 hours of release**					
**Rel in HCl**	0.85	0.87	0.98	0.86	0.86
**Rel in Phosphate**	0.84	0.86	0.95	0.79	0.87
**Applied for the first hour of release**					
**Rel in HCl buffer**	0.94	0.94	0.94	0.63	0.87
**Rel in Phosphate buffer**	0.997	0.996	0.91	0.39	0.996

Table S7. The calculated coefficient of determination (R^2) for the release kinetic of ibuprofen in phosphate buffer from **Zr-MOF2-IBU.**

Kinetic model	Zero Order	First Order	Higuchi	Korsmeyer-Peppas	Hixson–Crowell
**Applied for the first 5.66 hours of release**					
**Rel in Phosphate**	0.73	0.82	0.909	0.57	0.79
**Applied for the first hour of release**					
**Rel in Phosphate buffer**	0.995	0.999	0.948	0.16	0.999

Table S8. The calculated coefficient of determination (R^2) for the release kinetic in acidic or phosphate buffer for captopril using dialysis membrane from **Zr-MOF3-CP.**

Kinetic model	Zero Order	First Order	Higuchi	Korsmeyer-Peppas	Hixson–Crowell
**Applied for the first 1.5 (for acidic) respectively 5.66 (for alkaline) hours of release**					
**Rel in HCl**	0.999	0.69	0.999	0.32	0.895
**Rel in Phosphate**	0.857	0.946	0.95	0.698	0.92
**Applied for the first hour of release**					
**Rel in HCl buffer**	0.999	0.98	0.999	0.12	0.993
**Rel in Phosphate buffer**	0.999	0.998	0.92	0.24	0.999

Table S9. Examples of UIO-66 type materials used for drug loading and release

Type of material	Drug	Drug loading capacity	Method of analysis	Drug release	Method of analysis	Reference
-UiO-66-PDC	Ibuprofen sodium (IBUNa)	27.60 wt%<br>- UiO-66-PDC - 70 mg<br>- IBUNa solution 10 mg/mL, 14 mL at 60° C for 72 h.	^{1}HNMR	Phosphate Buffer Saline (PBS) solution with two different pH values (pH 2.0 and pH 7.4).	high performance liquid chromatography (HPLC).	72
-UiO-66 nano-porous	ciprofloxacin (CIP)	84 %<br>activated UiO-66 powder (0.1 g) was mixed with	Drug loading (%) = (CIP weight in sample/	PBS (phosphate buffer saline, pH: 7.4) and (acetate buffer, pH: 5.0) solutions	spectroscopy UV/Vis at 276 nm	73

		CIP solution (10 mL, 20 mg/mL	total weight of sample) × 100%			
-UiO-66<br>-UiO-66-NH$_2$<br>-UiO-66- COOH<br>-UiO-67<br>-Zr-NDC	5-Fluorouracil (5-FU)	UiO-66-COOH 0.40±0.03 g/g<br>UiO-66-NH2 0.44±0.06 g/g<br>UiO-66 0.28±0.05 g/g<br>UiO-67 0.28±0.03 g/g<br>Zr-NDC 1.30±0.05 g/g<br>5-FU (20 mg) and MOFs (10 mg) in 10 mL of ethanol were dispersed by sonication and stirred at 25° C for 6 h.	high performance liquid chromatography (HPLC)		high performance liquid chromatography (HPLC)	74
-chitosan (CS) modified Zr-NDC	fuorescein isothiocyanate (FITC)			4 mg of FITC-labeled CS was added into a 6-well culture plate, and 4 mL of artificial gastric juice		
UiO-66 nanoparticles (NPs)	dexamethasone (dex) and rhodamine B (RhB)	UiO-66 NPs at 1 mg/mL were postsynthetically loaded with fluorescent cargo (rhodamine B, denoted RhB) or dex via incubation of UiO-66 with RhB or dex at 37 °C and shaking at 1000 rpm for 24 h.		a subset of the loaded NPs (with a 1:1 incubation ratio and 1 mg/mL UiO-66) were re-dispersed into one of two media: PBS, pH 7.4, or ALF (Artificial Lysosomal Fluid)	The supernatants were analyzed via fluorescence to detect the amount of RhB released.	75
-UIO-66<br>-UIO-66-NH$_2$<br>-UIO-66-NO$_2$	ketoprofen	2.5 g of ketoprofen was dissolved in 20 ml of ethyl alcohol, and then 150 mg of MOFs carriers	High performance liquid chromatography (HPLC)	The drug release was studied by dispersing 20 mg of MOFs samples into 200 mL of phosphate buffer saline (PBS) at pH 7.4, temperature	5 mL of supernatant was taken out to calculate the drug release using by UV–	76

		were added in this solution		of 37 °C and stirred constantly for 72 h	vis (at 260 nm).<br><br>The level of ketoprofen loaded was determined from high performance liquid chromatography (HPLC).	
-UiO-66<br>-MOFs/ drug were loaded into the CMC/PEO/PU core-shell nanofibers (CMC/PEO/PU).	Doxorubicin and folic acid	10 mg/ml MOF-DOX and MOF-FA suspensions were sonicated into the core solution for 0.5 h and following was stirred for 3 h before electrospinning process	UV–Vis spectrophotometer at 361 and 485 nm, respectively.	in PBS under 100 rpm shaking speed and 37 °C under acidic pH (pH:5) and physiologic pH of 7.4.	UV–Vis absorbance measurement at 485 nm for DOX, and 361 nm for FA	77
UiO-66-NH2 (U) and its magnetic UiO-66-NH_2 form (MU)	Oxaliplatin (OX)	30 mg OX was dissolved in 6 mL water and 20 mg of U was added to the OX solution. The mixture was kept in the dark at 0° C to prevent OX from degrading and stirred for 48 h.	OX concentrations were measured by UV–Vis.	The as-prepared nanodrugs were dried, separately weighted in 5 mg portions, and poured in 1 mL of double distilled water with pH = 5.5, which incubated at 37°C.	OX concentrations were measured by UV–Vis	78
-UiO-66<br>-UiO-66-NH_2	5-fluorouracil (5-Fu)	5-Fu: 27 wt.% UiO-66-2-NH2 particles were dispersed in ethanol (10 mL) by the sonication and then the water of 5-Fu (10 mg/mL, 10 mL) was added	UV/Vis spectroscopy	5-Fu loaded nano-UiO-66 products (70 mg) were compacted by the pressure of 3 MPa to obtain a pellet (12 mm in diameter) and then sealed into a dialysis bag (molecular cut off 50 kD), which was then soaked in 500 mL of phosphate buffer solution (PBS 0.04 M, pH ¼ 7.4) at 37 ° C under continuous shaking	UV/Vis spectroscopy	79

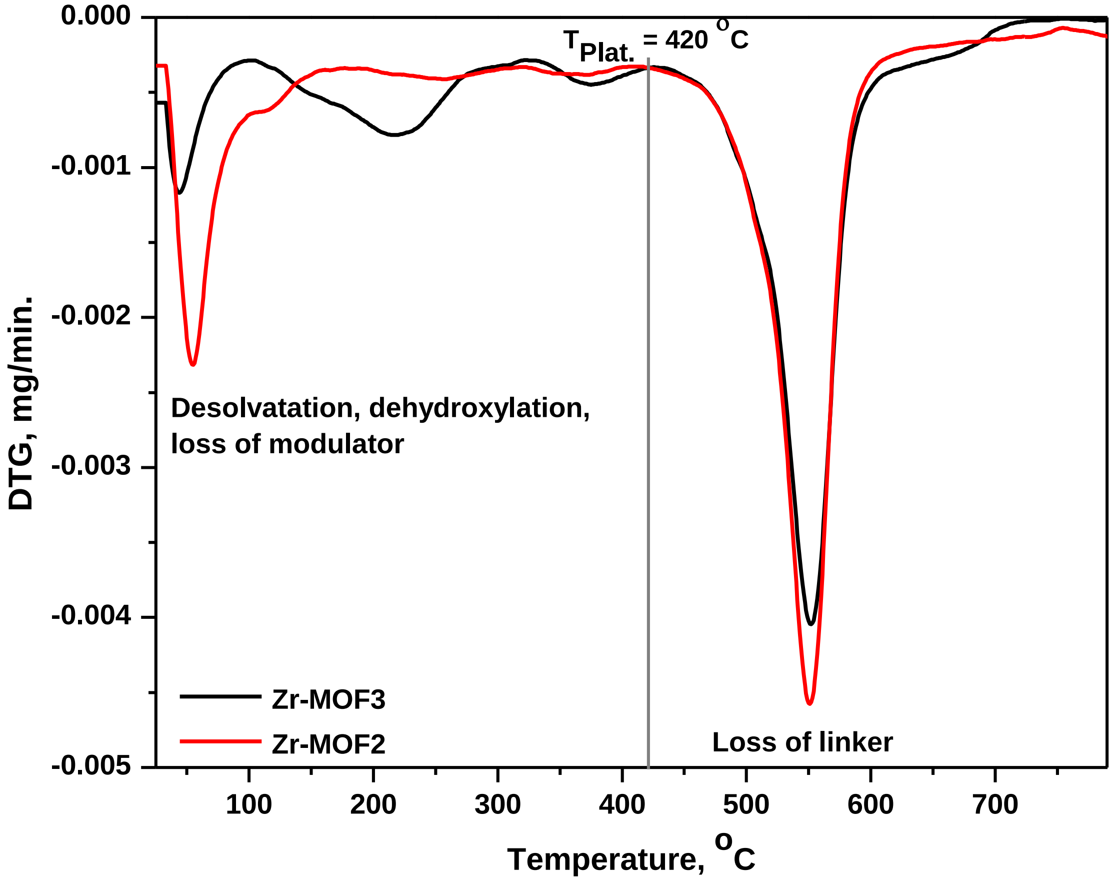

Figure S4. Derivative thermogravimetric curves of Zr-MOF2 and ZrMOF3

Energy dispersive X-ray spectrometry (EDX)

Energy dispersive X-ray spectrometry (EDX) was performed to map the elemental composition of the samples. As EDX spectra display, the main constituents of the samples are represented by: zirconium (Zr), carbon (C), oxygen (O). The drugs loading processes were done in 0.9% NaCl solutions, and the presence of Na and Cl in the **Zr-MOF2-IBU** and **Zr-MOF3-CP** is expected and justified. The appearance of nitrogen (N) can be explained by the presence of trace solvent (DMF) inside the pores of the materials.

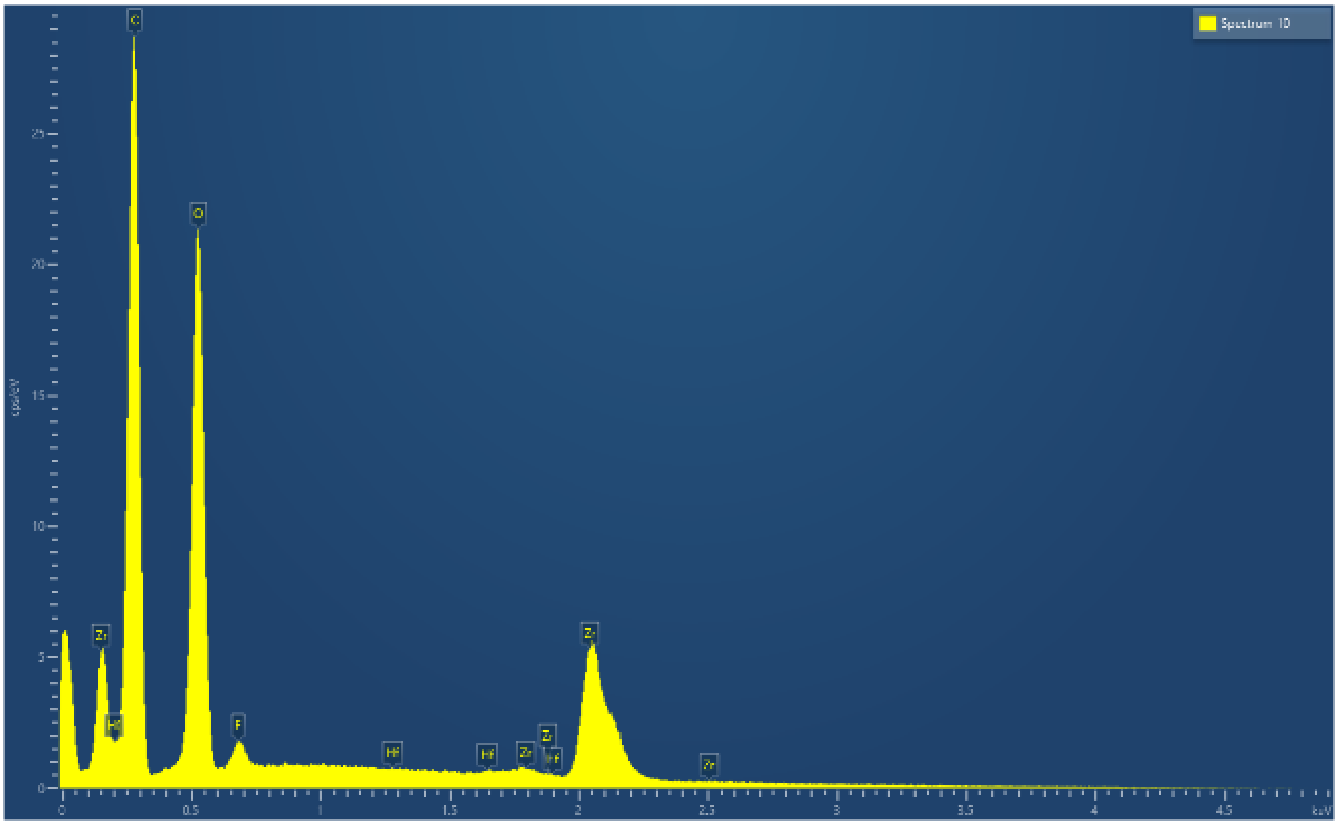

(a)

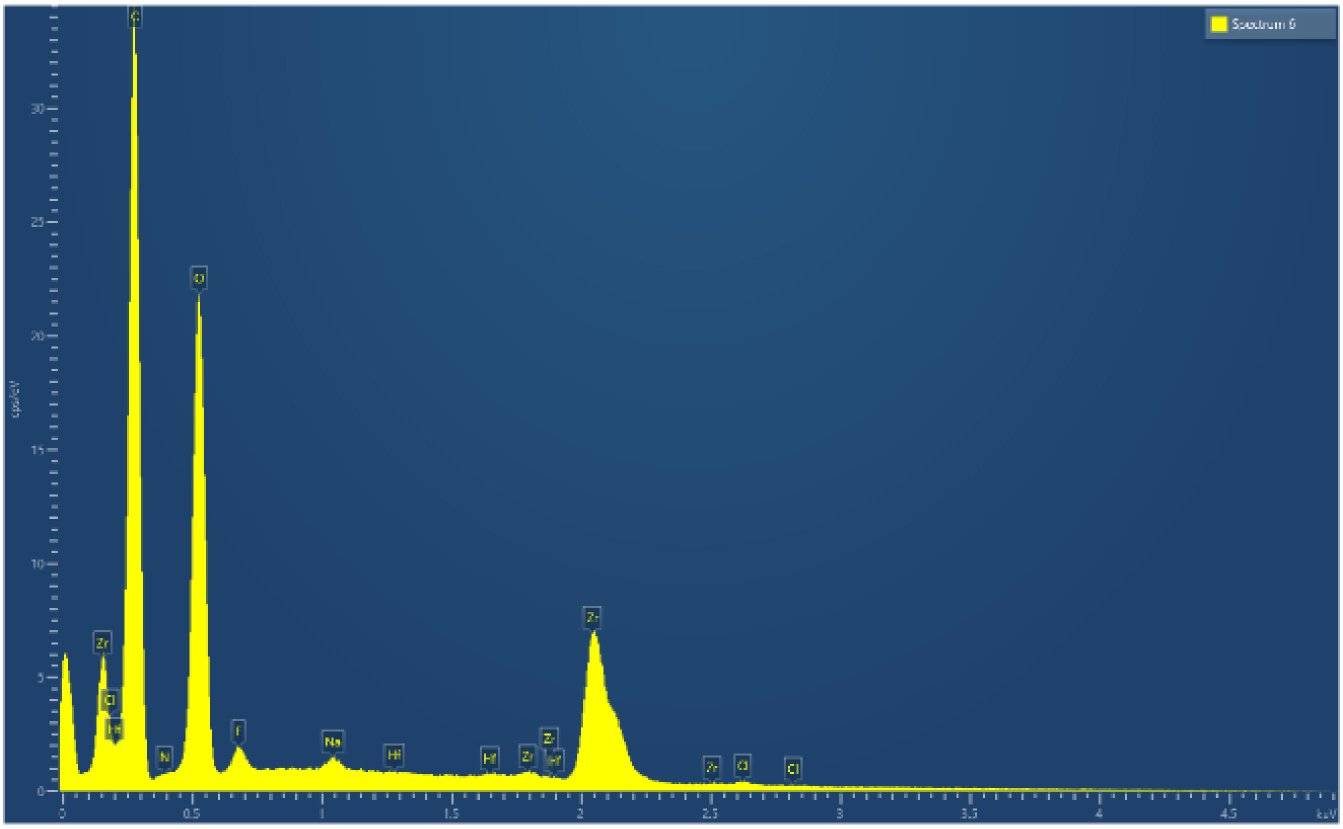

(b)

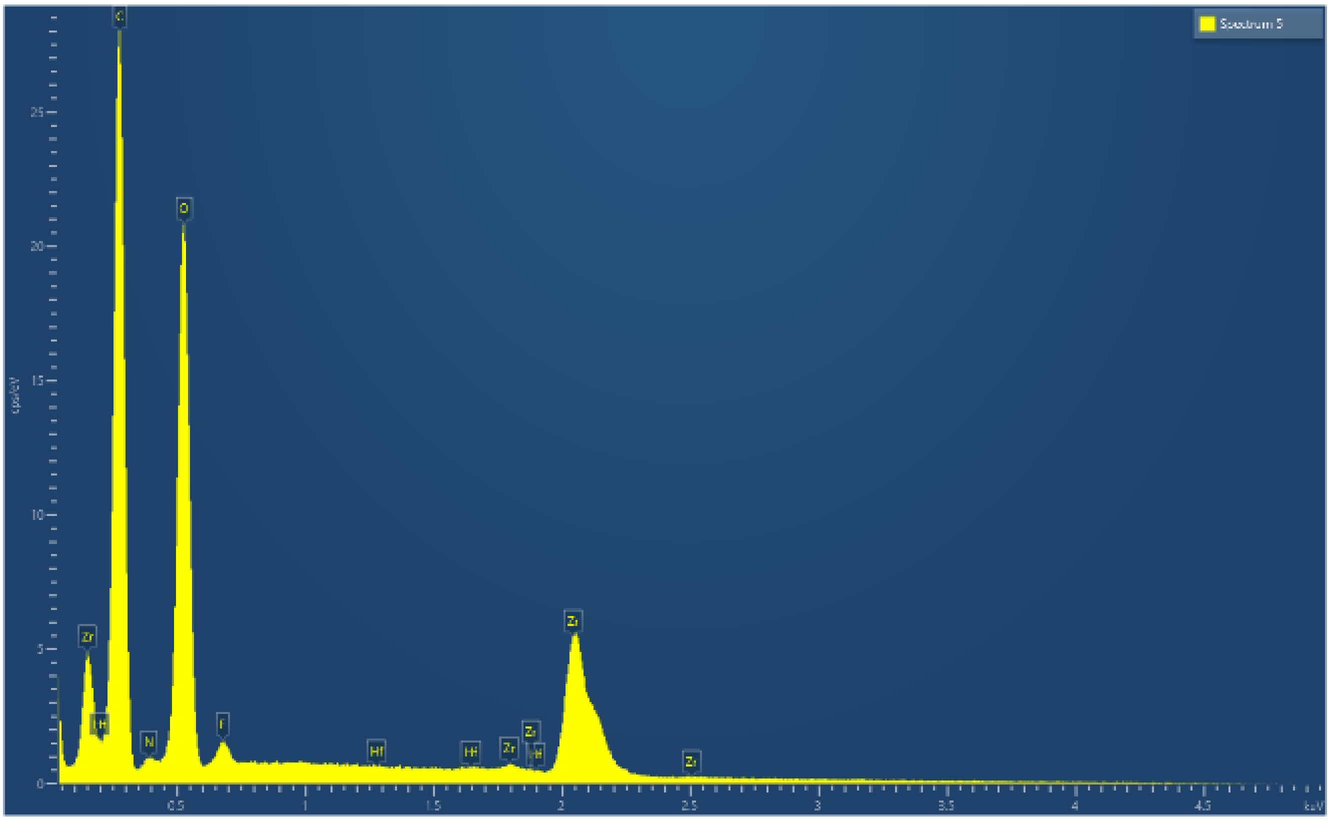

©

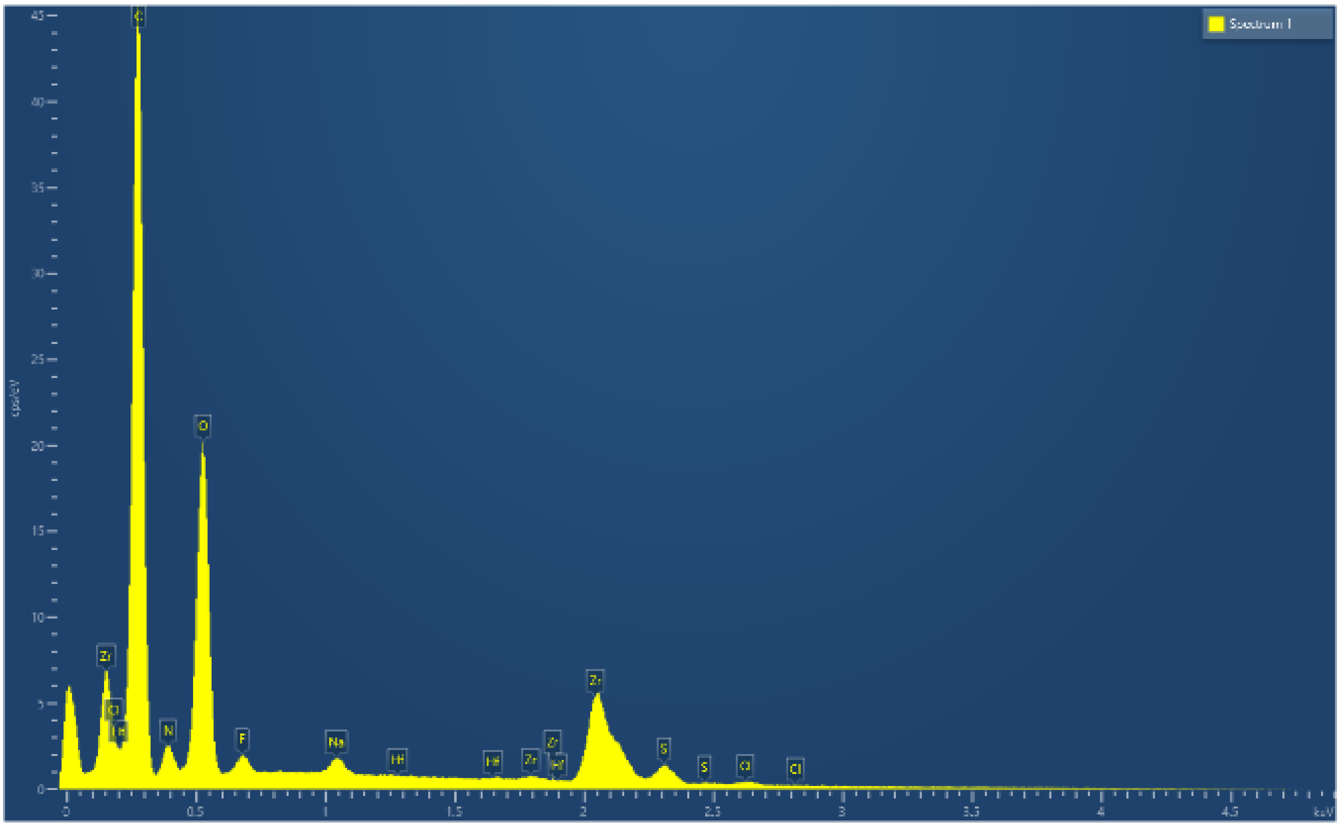

(d)

Figure S5. **EDX** spectra of the samples**:** (a) **Zr-MOF2**, (b) **Zr-MOF2-IBU**, (c) **Zr-MOF3** and (d) **Zr-MOF3-CP**.

References: 72 to 79 from Supplementary Material are cited into the main text.
